# Identification of bZIP Transcription Factors That Regulate the Development of Leaf Epidermal Cells in *Arabidopsis thaliana* by Single-Cell RNA Sequencing

**DOI:** 10.3390/ijms25052553

**Published:** 2024-02-22

**Authors:** Rui Wu, Zhixin Liu, Susu Sun, Aizhi Qin, Hao Liu, Yaping Zhou, Weiqiang Li, Yumeng Liu, Mengke Hu, Jincheng Yang, Jean-David Rochaix, Guoyong An, Luis Herrera-Estrella, Lam-Son Phan Tran, Xuwu Sun

**Affiliations:** 1 National Key Laboratory of Cotton Bio-Breeding and Integrated Utilization, School of Life Sciences, Henan University, 85 Minglun Street, Kaifeng 475001, China; wuruiwr347538@sina.com (R.W.); lzx2021@henu.edu.cn (Z.L.); su17861523158@sina.com (S.S.); qaz6835@henu.edu.cn (A.Q.); lhao0520@126.com (H.L.); zhouyapinghenu@sina.com (Y.Z.); weiqiangli@henu.edu.cn (W.L.); l13569882910@sina.com (Y.L.); mengkehu218511@sina.com (M.H.); 13458345215@sina.cn (J.Y.); angy@henu.edu.cn (G.A.); 2 State Key Laboratory of Crop Stress Adaptation and Improvement, School of Life Sciences, Henan University, 85 Minglun Street, Kaifeng 475001, China; 3 Departments of Molecular Biology and Plant Biology, University of Geneva, 1211 Geneva, Switzerland; jean-david.rochaix@unige.ch; 4 Institute of Genomics for Crop Abiotic Stress Tolerance, Department of Plant and Soil Science, Texas Tech University, Lubbock, TX 79409, USA; Luis.Herrera-Estrella@ttu.edu (L.H.-E.); Son.Tran@ttu.edu (L.-S.P.T.)

**Keywords:** bZIP transcription factors, jasmonic acid, pavement cells, scRNA sequencing, trichomes

## Abstract

Epidermal cells are the main avenue for signal and material exchange between plants and the environment. Leaf epidermal cells primarily include pavement cells, guard cells, and trichome cells. The development and distribution of different epidermal cells are tightly regulated by a complex transcriptional regulatory network mediated by phytohormones, including jasmonic acid, and transcription factors. How the fate of leaf epidermal cells is determined, however, is still largely unknown due to the diversity of cell types and the complexity of their regulation. Here, we characterized the transcriptional profiles of epidermal cells in 3-day-old true leaves of Arabidopsis thaliana using single-cell RNA sequencing. We identified two genes encoding BASIC LEUCINE-ZIPPER (bZIP) transcription factors, namely *bZIP25* and *bZIP53*, which are highly expressed in pavement cells and early-stage meristemoid cells. Densities of pavement cells and trichome cells were found to increase and decrease, respectively, in *bzip25* and *bzip53* mutants, compared with wild-type plants. This trend was more pronounced in the presence of jasmonic acid, suggesting that these transcription factors regulate the development of trichome cells and pavement cells in response to jasmonic acid.

## 1. Introduction

Epidermal cells are responsible for exchanging materials and information between the plants and the surrounding aerial environment [1]. In leaves, epidermal cells can differentiate and produce trichomes, which are a specialized cell type that protects plants from adverse conditions, including ultraviolet radiation and herbivore attack [2]. In addition to trichome cells (TCs), leaf epidermal cells are also composed of guard cells (GCs) and pavement cells (PCs) [3]. Previous studies have systematically and comprehensively characterized the developmental dynamics of the transcriptomes of stomatal lineage cells [4,5]. It is now important to examine the processes underlying the fates and development of PCs and TCs.

The irregularly zigzagged protrusions of leaf PCs are mainly regulated by the cytoskeleton [6]. The dynamic arrangement of microtubules plays a role in the development of PCs [7]. Microtubule-associated proteins KATANIN, IQ67 DOMAIN5 (IQD5), SPIRAL2, and CLASP are essential for the morphogenesis of PCs [8,9,10,11]. Microfilaments mainly control the outward projection of the edge of epidermal cells [12]. The Rho GTPase cascade signaling pathway is a foundation of the formation of PCs by activating microtubules and promoting their orderly arrangement which consequently leads to morphological changes of leaf epidermal cells [11].

Trichomes (TCs), emerging from the epidermis of aerial plant organs, represent an exemplary model for exploring cellular differentiation in plants [3]. Their distribution is characterized by regular spacing and a notable avoidance of adjacency, indicating a highly controlled spacing mechanism [13,14,15,16,17,18,19,20,21,22]. In Arabidopsis thaliana, over 40 genes have been linked to the initiation and differentiation of TCs, highlighting the complexity of this developmental process [23,24]. Critical to TC development is the interplay of regulatory transcription factors (TFs), necessitating both activator and repressor functions [25,26]. Among the activators, several TF families including MYB, BASIC HELIX–LOOP–HELIX (bHLH), WDR, and C2H2 ZINC FINGER, play significant roles. Notably, R2R3-MYB TFs such as GLABROUS1 (GL1) and its paralog MYB23 are integral to this group [23,25,27,28,29,30]. Conversely, the regulation of TC development also involves a suite of repressors, comprising at least seven MYB proteins: CAPRICE (CPC), TRIPTYCHON (TRY), ENHANCER OF TRY AND CPC1 (ETC1), ETC2, ETC3, TRICHOMELESS1 (TCL1), and TCL2, which perform overlapping functions in TC initiation and differentiation [31,32,33,34,35,36]. These insights underscore a finely tuned regulatory network governing TC development in Arabidopsis.

Phytohormones play important roles in regulating trichome development [37,38], among which jasmonic acid (JA) is the most critical hormone [39,40,41,42,43]. JA is part of a broader regulatory network where it functions alongside other phytohormones and signaling molecules to mediate epidermal cell fate determination and trichome development [44,45,46]. For example, the effect of NUCLEOREDOXIN 2 (AtNRX2) on trichome formation in response to JA was recently studied, and the results showed that AtNRX2 plays a central role in JA-mediated trichome formation in *Arabidopsis* [43]. JASMONATE ZINC FINGER INFLORESCENCE MERISTEM (ZIM)-domain (JAZ) proteins play important roles in regulating the signaling of JA. It was established that JA promotes trichome development by enhancing the degradation of the JAZ repressor proteins to limit their interaction with GL1 and EGL3/GL3 [47,48]. Moreover, the induction of trichome formation as a defense mechanism against herbivores illustrates the adaptive significance of JA-mediated pathways. JA signaling has been shown to upregulate the expression of key transcription factors, such as GLABRA3 (GL3), which are central to the epidermal cell fate determination and trichome development. This indicates a sophisticated level of developmental plasticity controlled by JA and its derivatives, linking environmental cues to morphological and physiological adaptations [44].

Interestingly, one study reported that *ETC3* is highly expressed in young stomatal cells and that its expression is under the control of SPEECHLESS (SPCH), which is highly expressed in early-stage meristemoid (EM) cells [5]. These results suggest that SPCH may activate genes that regulate trichome differentiation and that EM cells may act as the precursors for TC production [5].

The fate determination of TCs and PCs is tightly regulated at the cellular level [49], for which the precise underlying molecular mechanisms are still unclear. Single-cell RNA-sequencing (scRNA-seq) technology allows the analysis of transcriptional profiles of different types of cells and the identification of genes that are specifically expressed at different developmental and morphogenetic stages [4,50,51,52,53,54,55,56,57,58,59,60,61]. Very recently, Lopez-Anido et al. (2021) investigated the stomatal lineage cell fate commitment and differentiation in *Arabidopsis* leaves using scRNA-seq analysis [62]. However, there have been fewer studies on the development of PCs and TCs, especially in terms of scRNA-seq research. In fact, the development of PCs and TCs is crucial for leaf growth and adaptability to the environment. Analyzing the developmental regulatory mechanisms of these cells is of great significance for understanding the development and environmental adaptability of leaves [63]. Due to the fact that the epidermal hairs of *Arabidopsis* are mainly formed on the true leaves, in this study, we conducted an scRNA-seq analysis of 3-day-old true leaves of *Arabidopsis* wild-type plants to elucidate the mechanisms that regulate the fate and development of TCs and PCs. Our study identified a group of novel marker genes for PCs and TCs and discovered the new roles of two BASIC LEUCINE-ZIPPER (bZIP) TFs in the regulation of fate determination and differentiation of PCs and TCs through the comparative analysis of WT and single and double mutants of *bZIP25* and *bZIP53* genes.

## 2. Results

### 2.1. Single-Cell Transcriptional Profiling of Leaf Epidermal Cells Unravels Different Cell Types and Gene Expression Signatures

**Figure 1:**
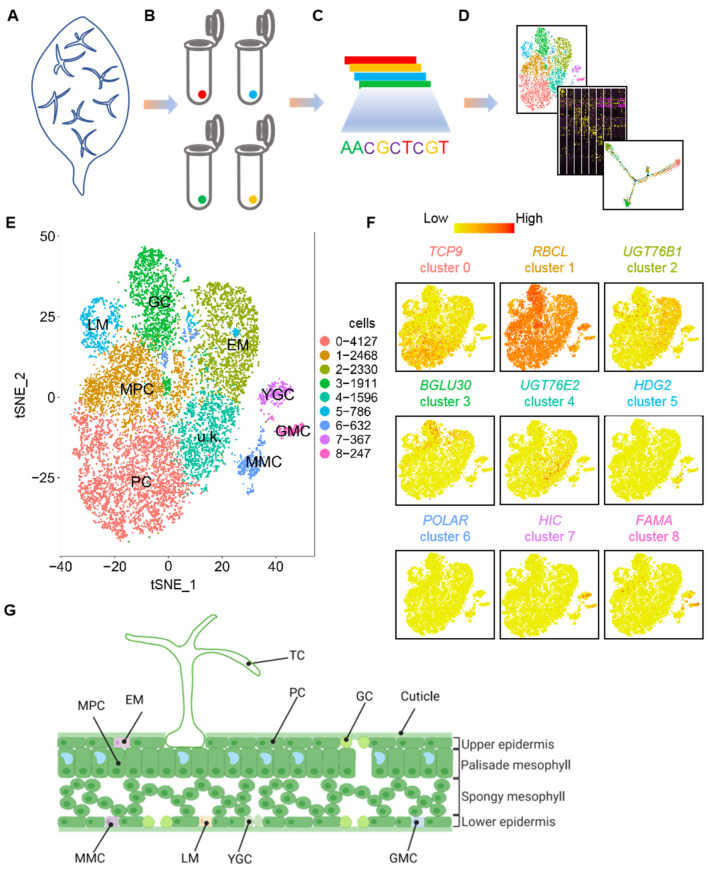
Distinct cell subpopulations with transcriptional signatures determined by single-cell RNA-sequencing analysis of epidermal cells of true leaves. (**A**–**D**) Illustration of the scheme used for young leaves (**A**), cell isolation (**B**), sequencing (**C**), and downstream analysis (**D**). (**E**) t-distributed stochastic neighbor embedding (tSNE) plot reveals cellular heterogeneity with 9 distinct clusters of cells identified and color-coded. The general identity of each cell cluster is defined in the corresponding cluster. (**F**) Feature plots of expression distribution for selected marker genes. Expression levels for each cell are color-coded and overlaid onto the tSNE plot. (**G**) Illustration of a leaf section with the different cell types. TC, trichome cell; EM, early-stage meristemoid; GC, guard cell; PC, pavement cell; LM, late-stage meristemoid; YGC, young guard cell; MPC, mesophyll cell; GMC, guard mother cell; MMC, meristemoid mother cell; u.k., unknown.

We subjected protoplasts of 3-day-old true leaves of *Arabidopsis* to scRNA-seq analysis to identify cell-type-specific changes in gene expression that occur during epidermal cell fate determination at a single-cell resolution (Figure 1A–D). A total of 18,000 cells were subsequently used to generate the libraries that were sequenced (Figure 1B,C). After stringent cell filtration, high-quality transcriptomes of 15,773 individual cells were retained for subsequent analyses (Figure 1D). A total of 512,130,798 reads were obtained after the sequencing data were processed, with an average of 32,468 reads and 2118 genes identified per cell. The percentage of reads mapped to the genome was 93%. We then performed a tSNE dimensionality analysis of the scRNA-seq data. Figure S1A,B illustrate the tSNE projection plots of cells colored by UMI counts and automated clustering, respectively.

**Figure 2:**
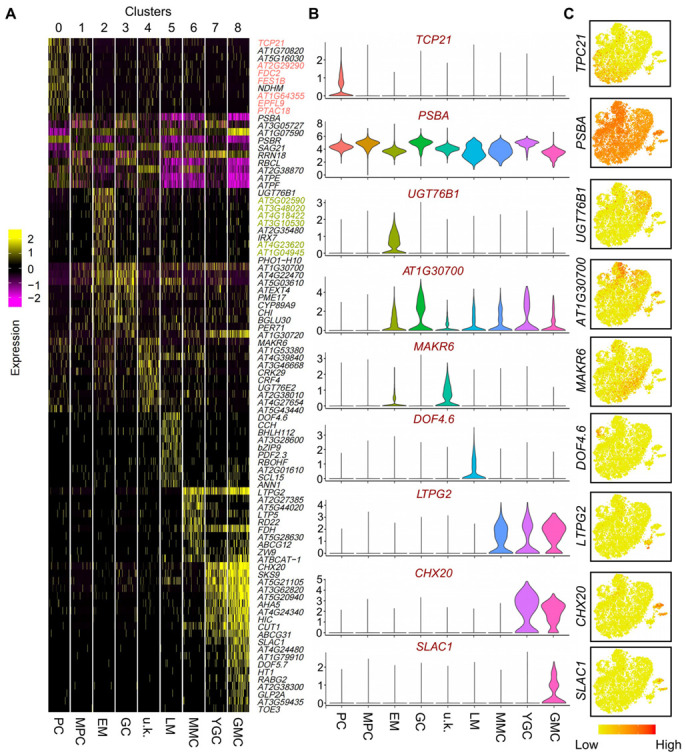
Identification of novel marker genes for each cluster. (**A**) Heatmap of differentially expressed genes (DEGs). The top 10 genes and their relative expression levels in all sequenced cells are shown for each cluster. The color ranges from purple to yellow and represents the expression value of the marker genes from low to high. (**B**) Violin plots of selected novel marker genes for each cluster. (**C**) Feature plots of the expression distribution of selected novel marker genes. Expression levels for each cell are color-coded and superimposed on the tSNE plot. EM, early-stage meristemoid; GC, guard cell; PC, pavement cell; LM, late-stage meristemoid; YGC, young guard cell; MPC, mesophyll cell; GMC, guard mother cell; MMC, meristemoid mother cell; u.k., unknown.

The sequencing saturation satisfied the requirement of 10× Genomics (Figure S1C). The median number of genes per cell (using TAIR10 as the reference genome) also met the requirement for data analysis (Figure S1D). We then analyzed the scRNA-seq data by PCA. Figure S1E shows the distribution of mitochondrial gene sequences (percent. mito) on a PCA plot. Figure S1F,G display the UMI distribution and number of nuclear-encoded genes [64] on the PCA plot. After removing mitochondrial transcripts, a total of 14,464 cells were used for the subsequent analysis. Subsequently, tSNE analysis was carried out on the selected cells. As shown in Figure 1E, nine cell clusters were identified as being independently distributed on the tSNE plot. A scheme for the distribution of the different cell types in the leaf cross-section is shown in Figure 1G. We also visualized cell clusters using the UMAP algorithm on our scRNA-seq data (Figure S2). The UMAP analysis produced similar cell clusters as those in the tSNE analysis, as shown by a Pearson correlation analysis (Figure S2A,B). On the leaf surface pattern diagram, we reported the distribution models of stomatal cells and PCs at different developmental stages (Figure S2C). We identified cell-type-specific expressed genes in the different cell types (Table S1). The expression patterns of the top 10 genes in each cell type are shown in a heatmap plot (Figure 2A). The violin plots and feature plots of representative cell-type-specific expressed genes in each cell type are shown in Figure 2B,C.

We then determined the cell type of the identified cell clusters using well-defined cell-type-specific marker genes. As shown in Figure 1F and Figure 2A–C, the epidermal marker gene for PCs, *TCP21* [4], was chiefly expressed in cluster 0; the marker gene for mesophyll cells (MPCs), *RIBULOSE BISPHOSPHATE CARBOXYLASE LARGE CHAIN* (*RBCL*) [4], was primarily distributed in cluster 1; the marker gene for EM cells, *UDP-DEPENDENT GLYCOSYLTRANSFERASE 76B1* (*UGT76B1*) [4], was mainly expressed in cluster 2; the marker gene for GCs, *BETA-GLUCOSIDASE* (*BGLU30*) [4], was predominantly distributed in cluster 3; the marker gene for LMs, *DNA BINDING WITH ONE FINGER 4.6* (*DOF4.6*) [4], was mainly enriched in cluster 5; the meristemoid mother cell (MMC) marker gene, *HOMEODOMAIN GLABROUS 2* (*HDG2*) [4], was chiefly expressed in cluster 6; the young guard cell (YGC) marker gene, *HIGH CARBON DIOXIDE* (*HIC*) [4], was mainly expressed in cluster 7; the GMC marker genes, *FAMA* and *DOF5.7* [4], were mostly expressed in cluster 8. However, the transcript of the marker gene for TCs, namely *GL2* [65], was unfortunately not detected in abundance in our scRNA-seq data, and *GL3* was only detected in some cells in EM cells and GMCs (Figure S3). Similar observations were also reported previously [5]. The lower detection of TCs’ marker genes was perhaps due to the size of TCs being too large to pass through the cell strainer. In addition, no known marker genes were identified as expressed in cluster 4. Collectively, our results indicate that cluster 0 belongs to PCs, cluster 1 to MPCs, cluster 2 to EM cells, cluster 3 to GCs, cluster 4 to unknown (u.k.) cells, cluster 5 to LM cells, cluster 6 to MMCs, cluster 7 to YGCs, and cluster 8 to GMCs. Notably, the expression levels of some marker genes of the JA signal transduction pathway, such as *ACYL-COA OXIDASE 1* (*ACX1*) [66], *ABNORMAL INFLORESCENCE MERISTEM* (*AIM1*) [67], *BLADE ON PETIOLE1* (*BOP1*) [68], *CORONATINE INSENSITIVE 1* (*COI1*) [69,70], *CONSTITUTIVE EXPRESSION OF PR GENES 5* (*CPR5*) [71], *CULLIN 1* (*CUL1*) [72], *JASMONATE-ZIM-DOMAIN PROTEIN 10* (*JAZ10*) [73], *JASMONATE-INDUCED OXYGENASE2* (*JAO2*), *JASMONATE-INDUCED OXYGENASE3* (*JAO3*) [74], *PRODUCTION OF ANTHOCYANIN PIGMENT 1* (*PAP1*) [75], *RADICAL-INDUCED CELL DEATH1* (*RCD1*) [76], and *RIBONUCLEASE 1* (*RNS1*) [77] were also remarkably high in cluster 2, suggesting that they may function in EM cells (Figure S4).

### 2.2. Selection and Characterization of Newly Identified Cell-Type-Specific Expressed Genes in PCs and EM Cells

GO analysis was then performed to identify the potential biological function of cell-type-specific expressed genes in each cell cluster (Table S2). As shown in Figure S5, GO terms in MPCs and PCs were generally very similar and different from those in the other cell types. GO terms in the u.k., LM, GMC, and EM clusters were comparable, suggesting that these genes are involved in similar biological processes in these different cell types. GO terms for MPCs were predominantly related to photosynthesis, consistent with their functions (Figure S5). Considering the high similarity in GO terms in clusters 3 and 7, we propose that cluster 3 belongs to GCs (Figure S5). In our previous study, we found that some marker genes were detected in several cell types but at different levels of gene expression [4]. The top 10 cell-type-specific expressed genes for each of the studied cell types other than TCs were specifically expressed in the corresponding cell types, except for the markers of MPCs, GMCs, and GCs. Some marker genes of PCs, such as *FERREDOXIN C 2* (*FDC2*), *FES1B*, *AT2G29290*, and *EPIDERMAL PATTERNING FACTOR LIKE-9* (*EPFL9*), were also enriched in MPCs and GCs (Figure 2A).

To investigate the expression patterns of identified cell-type-specific expressed genes, we constructed promoter-driven *GUS* reporter constructs for some of the representative genes to analyze their tissue-specific expression (Table S2). *AT2G29290*, *FES1B*, *TCP21*, *PLASTID TRANSCRIPTIONALLY ACTIVE 18* (*PTAC18*), *FDC2*, and *AT1G64355* were selected as representative genes for PCs, while *AT1G04945* and *EUKARYOTIC RELEASE FACTOR 1-2* (*ERF1-2*) were selected as representative genes for EM cells, and the corresponding transgenic plants were produced. We preferably selected *ERF1-2* because it was previously reported to be expressed in stomata [78], and we were curious whether it is expressed in EM cells of true leaves. GUS staining analysis revealed that the selected genes are expressed in the leaves of seedlings (Figure S6). Some genes, such as *TCP21*, *FDC2*, *ERF1-2*, and *AT1G04945*, are also expressed in roots (Figure S6), suggesting that these genes may also play important functions in root epidermal cells. To test whether the above-mentioned genes could be expressed in the PCs or EM cells, we performed a histological section analysis of some *GUS* transgenic lines, including *TCP21pro::GUS*, *AT1G64355pro::GUS*, *AT2G29290pro::GUS*, *AT1G04945pro::GUS*, *ERF1-2pro::GUS*, and *FES1Bpro::GUS*. As shown in Figure S7, GUS staining can be detected in MPCs and epidermal cells, including PCs and GCs, in these transgenic lines. *GUS* expression in these transgenic lines was also detected in MPCs. These results are consistent with our scRNA-seq data. Indeed, these genes, such as *TCP21*, *AT2G29290*, and *FES1B*, are also expressed in MPCs, as shown by scRNA-seq (Figure 2A).

**Figure 3:**
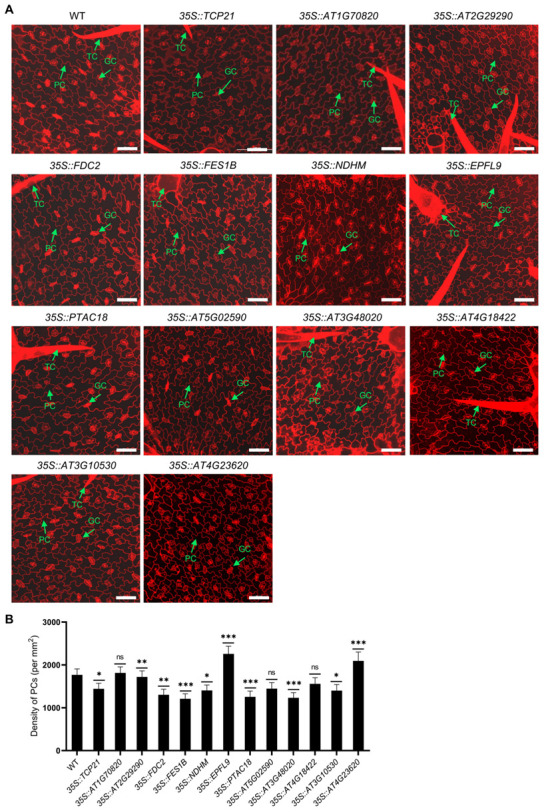
Characterization of the potential roles of selected marker genes for pavement cells (PCs). (**A**) Analysis of the developmental status of PCs in WT and transgenic lines. Samples were stained with propidium iodide, after which PCs were detected using a laser confocal microscope. Scale bar (50 μm) is shown as a white line. (**B**) The density of PCs in the upper epidermis of 3-day-old true leaves of WT and transgenic seedlings. Data are represented as mean ± SD (*n* = 3). Asterisks indicate a significant difference between transgenic and WT plants as determined using Student’s *t*-test. * *p* < 0.05, ** *p* < 0.01, and *** *p* < 0.001. ns, non-significant.

Transgenic plants were then successfully produced that overexpressed several selected newly identified PC-related (e.g., *TCP21*, *AT1G70820*, *AT2G29290*, *FDC2*, *FES1B*, *NDHM*, *EPFL9*, and *PTAC18*) and EM-related (e.g., *AT5G02590*, *AT3G48020*, *AT4G18422*, *AT3G10530*, and *AT4G23620*) genes to determine their potential roles in the regulation of PC and TC development (Table S2). The expression levels of target genes in corresponding transgenic lines were examined by qPCR (Figure S8). The results indicated that, compared with WT plants, the overexpression of *TCP21*, *FDC2*, *AT4G18422*, *AT3G10530*, and *AT4G23620* (PC-specific expressed genes) resulted in a significant decrease in the density of TCs, while that of *FES1B*, *NDHM*, and *EPFL9* (EM-specific expressed genes) enhanced the density of TCs (Figure S9A,B). PC densities were significantly lower, relative to WT plants, in seedlings of *35S::FES1B*, *35S::PTAC18*, *35S::FDC2*, and *35S::AT3G48020* transgenic plants, but significantly higher in *35S::EPFL9* and *35S::AT4G23620* transgenic seedlings (Figure 3A,B). These results suggest that the selected PC- and EM-specific expressed genes may be involved in the development of PCs and TCs. The identification of these new cell-type-specific expressed genes provides a reference for us to identify PCs and EM cells in future scRNA-seq studies.

### 2.3. Pseudo-Time Trajectory Analysis of the Spatiotemporal Dynamics of Epidermal Cell Differentiation

**Figure 4:**
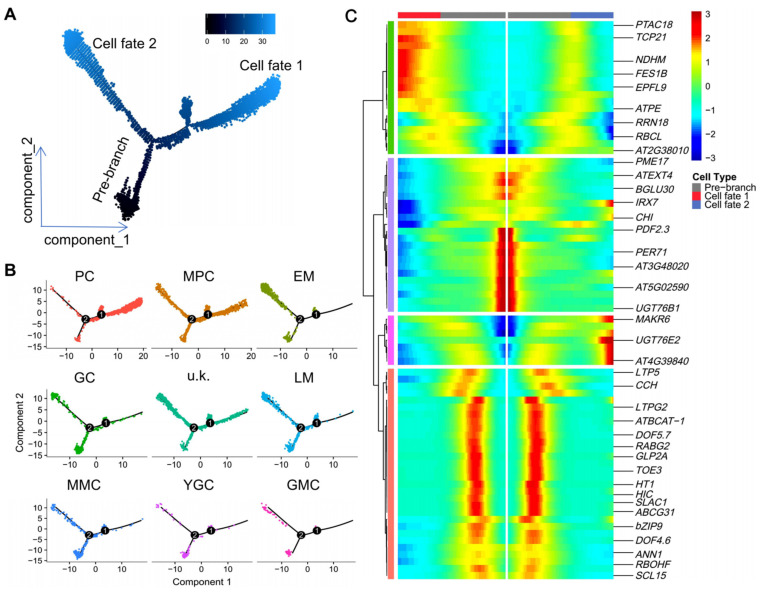
Pseudo-time analysis reveals putative differentiation trajectories of different cell types. (**A**) Distribution of cells of each cluster on the pseudo-time trajectory. (**B**) Distribution of cells of each cell type on the pseudo-time trajectory. (**C**) Clustering and expression kinetics of the top 10 genes in all clusters along with a pseudo-time progression (the representative marker genes were shown). EM, early-stage meristemoid; GC, guard cell; PC, pavement cell; LM, late-stage meristemoid; YGC, young guard cell; MPC, mesophyll cell; GMC, guard mother cell; MMC, meristemoid mother cell; u.k., unknown.

*Arabidopsis* leaf development is a strictly regulated process that ensures that almost all leaves have similar spatial morphological characteristics at the same developmental stage [79,80,81]. The spatiotemporal regulation of leaf development is closely related to that of cell development [82,83]. Therefore, understanding the spatiotemporal regulation pattern of cell development is important for understanding leaf development. Taking this into consideration, we performed a pseudo-temporal ordering of cells (pseudo-time) on the scRNA-seq data using Monocle 2 [84] to reconstruct the developmental trajectory during differentiation. The resulting pseudo-time path had two nodes and three branches (Figure 4A), and different cell clusters were arranged relatively clearly (except for MPC and u.k.) at different branch sites of the pseudo-time path (Figure 4B). A heatmap analysis based on pseudo-time results was then constructed to characterize the spatiotemporal dynamic patterns of the top 10 genes of each cluster. As shown in Figure 4C, the heatmap of several representative genes from each cluster indicated a positive correlation between their expression dynamics and their cell distribution on the developmental trajectory. For example, *UGT76B1* and *PEROXIDASE 71* (*PER71*) are maximally expressed in the pre-branch of the pseudo-time trajectory, while *TPC21* and *EPFL9* are mostly expressed in the late stage of cell fate 1 (Figure 4C). Marker genes of stomatal lineage cells, such as *HIC* and *DOF5.7*, have their highest expression levels in the early stage of cell development, while these genes are downregulated following the developmental direction of cell fate 1 and cell fate 2 (Figure 4C). These results indicate that genes expressed in different cell types have a specific spatiotemporal pattern on the pseudo-time trajectory.

### 2.4. Analysis of the Effects of JA on the Development of TCs and PCs

Since JA-signaling-related genes are expressed in EM cells (Figure S4), it is possible that JA could be involved in the regulation of EM differentiation. It has been proposed that EM cells give rise to both PCs and TCs [5], and JA plays important roles in regulating the development of TCs [41]. Therefore, to explore this possibility, we first analyzed the process of TC differentiation in WT seedlings in the presence of JA. The results indicated that the number of TCs significantly increased in the presence of 20 μM JA (Figure S10A,C). Higher concentrations of JA (>40 μM) inhibit leaf growth, although the density of TCs gradually increases with increasing JA dose (Figure S10B,C). We then analyzed the effects of JA on the development of PCs and found that the density of PCs decreased along with increasing JA concentrations (0 to 40 μM) (Figure S11).

### 2.5. bZIP TFs Are Involved in Regulating the Fate and Differentiation of PCs and TCs

**Figure 5:**
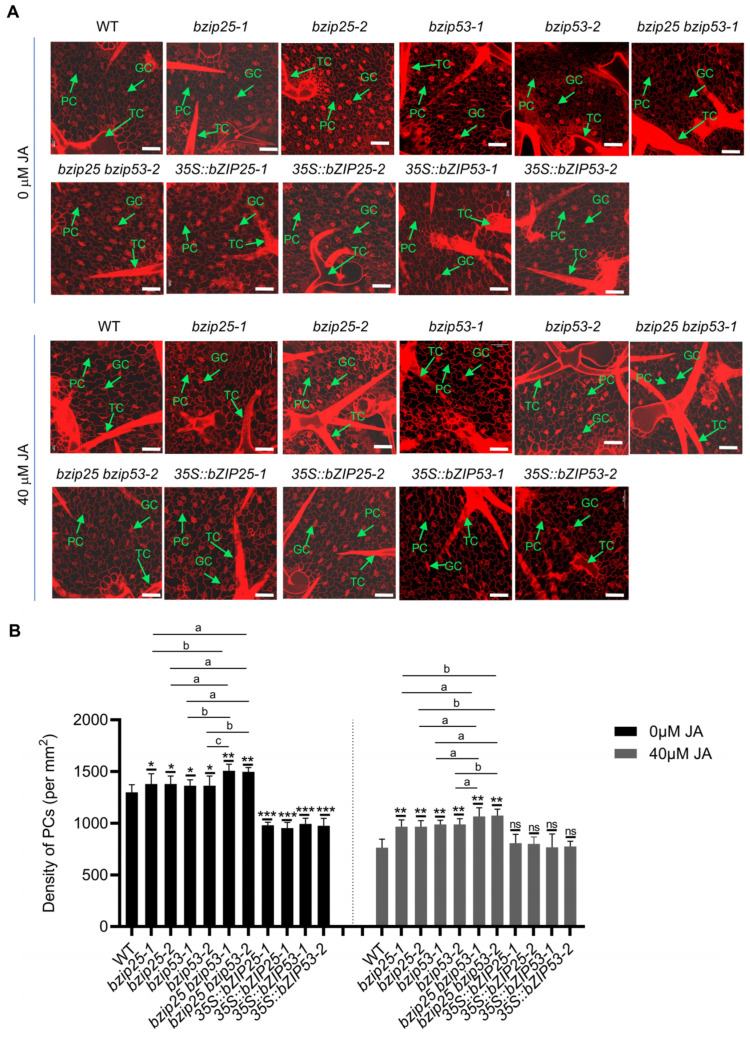
bZIP25 and bZIP53 negatively regulate pavement cell development. (**A**) Representative photographs of PCs in the upper epidermis of 3-day-old true leaves of WT, *bzip25-1*, *bzip25-2*, *bzip53-1*, *bzip53-2*, *bzip25 bzip53*, *35S::bZIP25*, and *35S::bZIP53* plants subjected to 0 (control) and 40 μM jasmonic acid (JA) treatments. The samples were treated with propidium iodide (PI) staining to show the cell wall. Bar, 50 μm. (**B**) The density of PCs in the upper epidermis of two 3-day-old true leaves of WT, *bzip25-1*, *bzip25-2*, *bzip53-1*, *bzip53-2*, *bzip25 bzip53*, *35S::bZIP25*, and *35S::bZIP53* plants subjected to 0 and 40 μM JA treatments. Data are represented as mean ± SD (*n* = 3). Asterisks indicate a significant difference between mutant and WT and between overexpression lines and WT as determined using Student’s *t*-test. * *p* < 0.05, ** *p* < 0.01, and *** *p* < 0.001. ns, non-significant. Letters indicate a significant difference between single mutant and double mutant as determined using Student’s *t*-test. ^a^ *p* < 0.05, ^b^ *p* < 0.01, ^c^ *p* < 0.001.

In our search for potential regulators of PCs and TCs, we identified two TF-encoding genes, *bZIP25* and *bZIP53,* that were predominantly expressed in EM cells and PCs (Figure S12A,B). In addition, because *bZIP25* and *bZIP53* encode TFs that regulate the expression of many other downstream genes and thus may play significant roles in regulating EM and PC development, these two genes were preferably selected for functional analysis in this study. *GUS* expression can be detected in the true leaves of *bZIP25pro::GUS* transgenic plants (Figure S13A). Notably, GUS signals in the *bZIP25pro::GUS* plants can be detected in these epidermal cell types (Figure S13B). We then examined the corresponding T-DNA insertion mutants *bzip25-1*, *bzip25-2*, *bzip53-1*, and *bzip53-2* to investigate the potential roles of bZIP25 and bZIP53 in the regulation of PC and TC development. The developmental states of TCs in leaves of the single *bzip25-1*, *bzip25-2*, *bzip53-1*, and *bzip53-2* mutant seedlings are shown in Figure S13C. The results indicated that the TC densities in *bzip25-1*, *bzip25-2*, *bzip53-1*, and *bzip53-2* seedlings were lower than those in WT plants with and without JA treatment, but the responses to JA decreased in the mutants as compared with that of the WT (Figure S12C,D). In contrast, the analysis of PCs showed that the PC densities in *bzip25-1*, *bzip25-2*, *bzip53-1*, and *bzip53-2* seedlings were higher than those in the WT with and without JA treatment (Figure 5A,B). We also generated the overexpression lines *35S::bZIP25* and *35S::bZIP53* to analyze the effects of higher expression of these genes on the development of TCs and PCs (Figure 5, Figures S12 and S14). Consistently, a greater number of TCs was observed in *35S::bZIP25-1*, *35S::bZIP25-2*, *35S::bZIP53-1*, and *35S::bZIP53-2* plants, while the number of PCs in *35S::bZIP25-1*, *35S::bZIP25-2*, *35S::bZIP53-1*, and *35S::bZIP53-2* plants was lower than that in the WT (Figure 5A,B). These results indicate that bZIP25 and bZIP53 play a positive role in determining the density of TCs and a negative role in the density of PCs.

Next, to test whether *bZIP25* and *bZIP53* function in the same regulatory pathway of TC and PC development, we generated the double mutants *bzip25 bzip53* using CRISPR/Cas9 technology (Figure 5A and Figure S15). Under the control conditions (no JA), the number of TCs was lower in the double mutants, while the number of PCs was greater, relative to the single mutants and WT plants (Figure 5A,B and Figure S12C,D). The effects of JA on TC and PC development were weak in the double mutants, relative to the single mutants and WT plants (Figure 5A,B and Figure S12C,D). To analyze whether the cDNA of *bZIP25* and *bZIP53* could complement the *bzip53* and *bzip25* mutants, respectively, we first analyzed the expression of these two genes in *bzip53* and *bzip25* mutants, respectively. As shown in Figure S16, the expression level of *bZIP25* in the *bzip53* mutant was higher than that in WT plants, suggesting that the mutant plants try to rescue the loss of function of *bZIP53* by increasing the expression level of *bZIP25*. However, the expression level of *bZIP53* in *bzip25* mutant is lower than that in the WT. Therefore, in order to detect whether the overexpression of *bZIP53* in the *bzip25* mutant could complement the defect caused by the loss of function of *bZIP25*, we overexpressed *bZIP53* in the *bzip25* mutant background to produce the transgenic plant *35S::bZIP53 bzip25*. An analysis of the densities of PCs and TCs indicated that, compared with *bzip25*, the densities of PCs and TCs in *35S::bZIP53 bzip25* seedlings were restored to the WT levels (Figure S16C,D). Additionally, the expression levels of *bZIP25* and *bZIP53* significantly decreased in leaves of the WT plants after JA treatment (Figure S17), suggesting that JA might regulate the development of TCs and PCs by negatively regulating the expression of *bZIP25* and *bZIP53* genes. These results collectively suggest that *bZIP25* and *bZIP53* may play additive or partially redundant roles, and that *bZIP53* may have a more important role than *bZIP25* in regulating the fate and differentiation of PCs and TCs (Figure S18).

## 3. Discussion

Environmental elements such as light, temperature, and moisture play pivotal roles in shaping the development of trichomes [85] and stomata, which are vital for plant adaptation and survival [86,87]. The density of trichomes is influenced by light intensity, with increased illumination promoting their formation as a protective measure against herbivory and ultraviolet radiation [85]. Temperature variation influences the developmental pathways of both trichomes and stomata, enhancing the plant’s capacity to manage thermal regulation and moisture retention [86,87]. Furthermore, water availability directly affects stomatal density and functionality, enabling plants to modulate their transpiration rates in response to drought conditions [86]. These environmental stimuli are seamlessly integrated with hormonal signals, allowing plants to adeptly adjust their epidermal features to navigate fluctuating external conditions, thus optimizing their growth, gas exchange capabilities, and defensive strategies [86,87]. In particular, JA signaling plays a crucial role in regulating trichome formation and stomatal development in flowering plants [88]. The synergy between gibberellin (GA) and JA signaling regulates trichome development [89]. DELLAs and JAZ proteins interact with the WD-repeat/bHLH/MYB complex to mediate this synergistic action, indicating a complex network involving multiple hormonal pathways in trichome initiation [89]. MYC2, a key regulator in the JA signaling pathway, controls various aspects of plant defense and development, including trichome formation [90]. This transcription factor coordinates JA-mediated defense responses and regulates crosstalk between JA and other phytohormones, affecting trichome development under stress conditions [90]. JA and MYC transcription factors negatively regulate stomatal development in Arabidopsis cotyledons [91]. This regulation involves a reduction in stomata number upon treatment with methyl jasmonate, highlighting JA’s role in modulating stomatal development in response to environmental cues [91]. JA signaling interacts with other hormonal pathways, such as gibberellin and ethylene, to regulate plant growth, development, and defense [92]. This crosstalk is essential for the fine-tuning of JA-dependent processes, including trichome and stomatal development [92]. Our results indicated that genes related to the JA signaling pathway are specifically expressed in epidermal cells. Further analysis revealed that JA treatment can also inhibit the differentiation of PCs, reduce the density of PCs, and increase the size of PCs (Figure S11). These findings further expand the diversity of JA’s regulatory effects on the development of epidermal cells. Taken together, these findings underscore the importance of JA in regulating trichome and stomatal development and stress response mechanisms.

Utilizing scRNA-seq technology, we constructed the global landscape of the transcriptomes of young epidermal cell types in *Arabidopsis* leaves. Unlike cotyledons, leaf epidermal cell types are more complex, with the development of TCs as their most striking feature. The fate determination and differentiation of TCs are tightly regulated by both internal factors, such as hormones, and external cues, such as invading pests and pathogens [25,37,93,94]. The transcriptome of TCs has been extensively characterized but not at the single-cell level [23,95]. Also, no reports on epidermal cells at single-cell resolution are available for true leaves. TCs are differentiated from protodermal cells or EM cells [5]. Therefore, a comprehensive study of the transcriptomes of various epidermal cell types in true leaves will enable us to identify the potential key regulators of their differentiation and development.

Because EM cells, PCs, and TCs of true leaves interact with each other during differentiation and development [5], we can identify the regulatory factors regulating TCs by analyzing the key regulatory factors in EM cells and PCs. In the scRNA-seq data obtained in this study, we did not identify the cell type in which the well-known TC marker gene *GL2* is specifically expressed (Figure 1). One possible explanation is that the size of TCs is too large, and therefore, they were filtered out during the process of cell filtration used to prepare protoplasts for scRNA-seq. Thus, in this work, we mainly focused on the characterization of the transcriptomes of EM cells and PCs. We identified several genes that were specifically expressed in EM cells and PCs (Figure 2) and verified the cell types identified by studying the expression patterns of several representative marker genes in PCs and EM cells (Figure S6). Since EM cells and PCs are distributed along the entire upper epidermal layer of true leaves, *GUS* expression, which was controlled by the promoters of the marker genes of EM cells and PCs, occurred in all epidermal cells (Figure S6). The expression patterns of the examined cell-type-specific expressed genes in PCs and EM cells suggested that these genes may be involved in mediating the development of these two cell types (Figure S6). Our results also demonstrated that *FES1B* negatively regulates the development of PCs, while *EPFL9* and *AT4G23620* regulate PC development in a positive manner (Figure 3). At present, the distinction between PCs and EM cells is difficult due to the inability to define specific marker genes. Our results provide important data that can be used for identifying PCs and EM cells in future scRNA-seq studies of epidermal cell development.

GCs, PCs, EM cells, and TCs are the main cell types present in the upper epidermis of leaves of *Arabidopsis*. PCs, EM cells, and GCs differentiate from MMCs. According to the distribution of cells in the constructed pseudo-time trajectories, MMCs mainly appear at the initial stage, while EM cells and GCs are distributed over the later stages of pseudo-time trajectories (Figure 4B). This is consistent with the viewpoint that GCs and EM cells differentiate from MMCs [4]. The formation of TCs was highly similar to that of EM cells with regard to developmental regulation [5]. Therefore, the results of the pseudo-time trajectory of EM cells also support the evidence that EM cells or TCs are differentiated from MMCs. Pseudo-time heatmap analysis of the top 10 genes further confirmed this premise (Figure 4C). Our results indicate that the analysis of the spatiotemporal patterns of gene expression in specific types of cells significantly contributes to the understanding of their development.

The identification of key TFs in specific cell types can assist in the identification of important regulatory factors involved in the fate determination and development of specific cell types. *bZIP25* and *bZIP53* were identified in our analysis of TF-encoding genes that showed increased expression levels in PCs and EM cells (Figure S12A,B). ABI3 was reported to be involved in the heterodimer complex, probably by its interaction with bZIP10 and/or bZIP25 [96]. However, bZIP53 does not interfere with the interaction between ABI3 and bZIP10 or bZIP25 [96]. Interestingly, further studies showed that bZIP53 can form a heterodimer with bZIP25 to regulate seed maturation [97]. In addition, bZIP53 was found to be involved in regulating the diurnal adjustment of amino acid metabolism and metabolic reprogramming during salt stress responses [98,99]. bZIP53 also plays important roles in regulating germination and seedling establishment [100,101]. These studies suggested that bZIP25 and bZIP53 can function together in regulating some developmental processes. Previous studies have shown that JA promotes the development of TCs [41], but inhibits the development of leaves [102]. Our results demonstrate that high concentrations of JA inhibit leaf growth, but that TC density gradually increased with the increasing doses of applied JA (Figure S10B,C). Further analysis of the effects of JA (0 or 40 μM JA) on the developmental status of both PCs and TCs in the seedlings of the *bzip25* and *bzip53* single and double mutants revealed that *bZIP25* and *bZIP53* may have additive or partially redundant functions in the regulation of development of PCs and TCs (Figure S12). In summary, our results provide new insights into the mechanisms underlying the highly complex yet orderly orchestrated process of epidermal cell development. These findings provide a basis for further studies of novel regulators of specific cell types in the epidermis of leaves.

In conclusion, our research delves into the complex regulation of leaf epidermal cell development in Arabidopsis thaliana, highlighting the critical roles of bZIP transcription factors—especially bZIP25 and bZIP53—in directing cell differentiation in response to jasmonic acid signaling pathways. This investigation sheds light on the intricate transcriptional networks involved, thereby enriching our comprehension of plant developmental biology. The application of single-cell RNA sequencing has been pivotal in uncovering cellular diversity and regulatory mechanisms, representing a notable leap forward in the field of plant science. While our discoveries provide fresh perspectives, they also emphasize the necessity for further studies on the interactions of these transcription factors within more extensive signaling networks and their impact on plant stress responses.

## 4. Materials and Methods

### 4.1. Screening and Verification of Mutants

*Arabidopsis thaliana* (Col-0 ecotype) WT plants were used in the scRNA-seq experiments. Seeds were sterilized in 5% sodium hypochlorite and germinated on vertical, half-strength Murashige and Skoog (1/2 MS) plates. T-DNA-insertion *bzip25-1*, *bzip25-2*, *bzip53-1*, and *bzip53-2* mutants (SALK_119931, SALK_148423, SALK_069883, and SALK_078494, respectively) were obtained from the *Arabidopsis* Biological Resource Center (ABRC) (Table S3). Mutant lines homozygous for the T-DNA insertion were identified by PCR analysis using gene-specific and T-DNA-specific primers (Table S4 and Figure S19). All mutants and WT plants were grown in a climate chamber at 22 °C and 100 µmol photons m^−2^ s^−1^ under a 14 h light/10 h dark regime. In the experiments designed to examine the effect of JA on TC development, 3-day-old seedlings were treated by spraying them with methyl jasmonate (392707, Millipore Sigma, St. Louis, MO, USA). The seedlings were then placed in a sealed transparent plastic container and grown for 24 h. Then, the developmental status of TCs was photographically documented.

### 4.2. Constructs for Plant Transformation

#### 4.2.1. YFP-Fusion Expression Constructs

Full-length cDNA fragments of selected genes (*AT1G70820*, *PTAC18*, *AT3G10530*, *EPFL9*, *AT4G18422*, *AT4G23620*, *AT5G02590*, *bZIP25*, *bZIP53*, *FES1B*, *NDHM*, *TCP21*, *AT2G29290*, *FDC2*, *AT3G48020*) were PCR-amplified using the primer pairs described in Table S4. The resulting PCR products were purified and cloned into pDNOR201 by BP Clonase reactions (GATEWAY Cloning; Invitrogen, Waltham, MA, USA) according to the manufacturer’s instructions to generate pDONR-cDNA vectors. The resulting plasmids were then recombined into pB7YWG2.0 using LR Clonase reactions to generate the final constructs.

#### 4.2.2. GUS Reporter Constructs

The upstream 2000 bp fragments of selected genes (*AT1G64355*, *AT2G29290*, *PTAC18*, *bZIP25*, *ERF1-2*, *FES1B*, *TCP21*, *AT1G04945*, *FDC2*) were PCR-amplified using the primer pairs described in Table S2. The resulting PCR products were purified and cloned into pDNOR201 by BP Clonase reactions according to the manufacturer’s instructions to generate pDONR-cDNA vectors. The resulting plasmids were recombined into pBGWFS7 using LR Clonase reactions to generate the final constructs. The resulting reporter constructs were then used to detect the expression of GUS under the control of the promoters of different marker genes.

#### 4.2.3. CRISPR/Cas9 Constructs

sgRNA was designed for each gene (*bZIP25* and *bZIP53*) using the CRISPR-P server (http://cbi.hzau.edu.cn/cgi-bin/CRISPR, accessed on 1 January 2022). The sgRNAs were cloned and assembled in pBE1.1 using the Golden Gate cloning system with the primers (Table S4).

### 4.3. Plant Transformation

YFP-fusion expression and *GUS* reporter constructs were transformed into *Agrobacterium tumefaciens* strain GV3101 via electroporation. *A. tumefaciens* containing the different constructs were introduced into WT plants as described by Zhang et al., 2006 [103]. Arabidopsis wild-type seedlings, once reaching 3-4 weeks of age and displaying blooming inflorescences, were primed for Agrobacterium inoculation. This process involved introducing a measured quantity of Agrobacterium, harboring a recombinant plasmid, into 5 mL of YEP liquid medium supplemented with appropriate antibiotics. The culture was then incubated at 28 °C and agitated at 200 rpm for 12–16 h, or until the OD600 value attained a range of 1.2–1.6. Subsequently, the bacteria were transferred to centrifuge tubes and spun at 4000 rpm at room temperature for 20 min. Post-centrifugation, the supernatant was discarded, and the bacterial pellet was resuspended in a freshly made 1/2 MS inoculation solution, with the OD600 being adjusted to a target range of 0.6–0.8. A 0.03% (*v*/*v*) concentration of surfactant L-77 was then incorporated into the adjusted bacterial suspension. Following thorough mixing, the entire Arabidopsis inflorescence was submerged in the Agrobacterium suspension for approximately 30 s. The inoculated seedlings were then placed in a plastic tray, which was sealed with plastic film to preserve soil moisture, and stored in the dark for 24 h. The following day, the film was removed, and the seedlings were relocated to a growth chamber with standard lighting conditions to continue growth and await seed collection.

The resulting T1 transgenic plants were selected using BASTA as described previously [104]. Two independent homozygous transgenic lines were used in all experiments. The positive lines for the *bzip25 bzip53* double mutant (produced by CRISPR/Cas9) were screened based on hygromycin resistance (100 μg mL^−1^) and genomic DNA sequencing. Two independent homozygous T2 lines were used for further experiments. To generate the transgenic plant *35S::bZIP53 bzip25-1*, the *bZIP53* cDNA was introduced into the *bzip25-1* mutant background by transforming it with the *35S::bZIP53-YFP* vector. Two independent homozygous T2 lines were used for further experiments.

### 4.4. Sample Collection and Protoplast Preparation

Three-day-old true leaves were harvested and used to isolate protoplasts as previously described with slight modifications in the use of young leaf tissues [4,105]. The leaves were harvested from seedlings and cut into 2 mm sticks and submerged in a solution (0.5 mM CaCl_2_, 0.5 mM MgCl_2_, 5 mM MES, 1.5% Cellulase RS, 0.03% Pectolyase Y23, 0.25% BSA, actinomycin D [33 mg L^−1^], and cordycepin [100 mg L^−1^], pH 5.5) by vacuum infiltration for 10 min. The samples were then incubated for 3 h to isolate protoplasts. Afterward, the isolated protoplasts were washed three times with 8% mannitol buffer to remove Mg^2+^. Cells were then filtered with a 40 µm cell strainer. Cell activity was detected by trypan blue staining, and cell concentration was measured with a hemocytometer. The protoplasts with more than 90% activity rate were used for scRNA-seq library construction.

### 4.5. ScRNA-seq Library Preparation

The scRNA-seq libraries were prepared using a Chromium Single Cell 3′ Gel Beads-in-emulsion (GEM) Library & Gel Bead Kit v3 according to the manufacturer’s instructions (10× Genomics, Pleasanton, CA, USA). Gel Beads-in-emulsion (GEMs) were produced by merging barcoded Single Cell 3′ v3.1 Gel Beads with a Master Mix that contains cells, and Partitioning Oil, utilizing the Chromium Next GEM Chip G. Upon the formation of GEMs, the Gel Bead dissolved, releasing primers and lysing any encapsulated cells. These primers were then combined with the cell lysate and a Master Mix enriched with reverse transcription (RT) reagents. The incubation process enabled the generation of barcoded, full-length cDNA from poly-adenylated mRNA. Following this step, the GEMs were disrupted, and the pooled fractions were collected. The first-strand cDNA was purified from the resultant GEM-RT reaction mixture, which contained residual biochemical reagents and primers, using silane magnetic beads. This barcoded, full-length cDNA underwent PCR amplification to produce a sufficient quantity for library preparation. The cDNA amplicons were then enzymatically fragmented and size-selected to refine the amplicon size optimally. The resulting libraries incorporated the P5 and P7 primers, necessary for Illumina amplification. Consequently, a Chromium Single Cell 3′ Gene Expression Dual Index library features standard Illumina paired-end constructs, marked by the initiation and conclusion with P5 and P7 sequences.

### 4.6. ScRNA-seq Data Preprocessing

The raw data were processed as previously described [4]. The Cell Ranger pipeline (version 3.0.0) provided by 10× Genomics was used to demultiplex cellular barcodes and map reads to the TAIR10 reference genome. The unique molecular identifier (UMI) count matrix was processed using the R package Seurat (Version 2.3.4). To remove low-quality cells and multiple captures, further criteria were applied to filter out cells with UMI/gene numbers outside the limit of the mean value ± 2 standard deviations, assuming a Gaussian distribution of each cell’s UMI/gene number, following visual inspection of the distribution of cells by the fraction of mitochondrial genes expressed. After the critical filtering process, 14,464 out of 15,773 cells were retained for downstream analysis. The median value of the mapping rate was 66.8%, and the median number of genes detected in each cell was 2118. Library size normalization was performed in Seurat on the filtered matrix to obtain normalized counts.

### 4.7. Clustering Analysis of scRNA-seq Data

Genes with the greatest variable expression among single cells were identified using the method previously described [106]. The *t*-distributed stochastic neighbor embedding (tSNE) analysis, uniform manifold approximation and projection (UMAP) analysis, and identification of cell-type-specific expressed genes were performed as previously described [4,50]. Briefly, the average expression and dispersion were calculated for all genes, which were subsequently placed into 9 bins based on their expression. Principal component analysis (PCA) was performed to reduce the dimensionality on the log-transformed gene-barcode matrices of the most variable genes. Cells were clustered via a graph-based approach and visualized in 2 dimensions using *t*SNE. A likelihood ratio test, which simultaneously tests for changes in mean expression and percentage of cells expressing a gene, was used to identify cell-type-specific expressed genes in each cluster. In addition, we performed a UMAP analysis [107] to confirm the identification of cell clusters by *t*SNE. For PCA, the scaled data were reduced into 30 approximate PCs depending on the 6520 highly variable genes (set npcs = 30). Clusters were identified using the Seurat function ‘FindClusters’ with “resolution = 0.4”. The data structures were separately visualized and explored by UMAP (the ‘RunUMAP’ was run with “n.neighbors = 30, metric = correlation and min.dist = 0.3”).

### 4.8. Pseudo-Time and Trajectory Analysis

Pseudo-time trajectory analysis of single-cell transcriptomes was conducted using Monocle 2 [84] as previously described [4].

### 4.9. RNA Extraction and qRT-PCR

Total RNA was extracted with fastpure plant total RNA extraction kit (Cat. No. DC104, Vazyme; Nanjing, China). Total RNA was treated with DNaseI (Vazyme; Nanjing, China) for 30 min to remove the remaining DNA. The cDNA was synthesized with HiScript II One-Step RT-PCR Kit (Cat. No. P611, Vazyme; Nanjing, China), and qRT-PCR was performed with the corresponding primers (Table S4).

### 4.10. GUS Staining and Histological Analysis

Histochemical GUS staining was performed with a G3061 GUS staining Kit (Solarbio Co., Beijing, China) according to the manufacturer’s instructions as previously described [50]. Semithin sections were prepared according to the protocol described previously [108]. Subsequently, the semithin sections were scanned on Pannoramic MIDI FL (3D HISTECH, Budapest, Hungary).

### 4.11. Microscopy

Seedlings were stained with 10 μg mL^−1^ propidium iodide (PI) (P4170, Sigma, St. Louis, MO, USA) for 1 min prior to imaging. PI staining was used to stain the cell walls of epidermal cells. Fluorescence in roots was detected using a Zeiss LSM980 confocal laser scanning microscope (Zeiss, Oberkochen, Germany). The PI signal was visualized at 610 to 630 nm wavelengths.

### 4.12. Gene Ontology (GO) Enrichment Analysis

GO enrichment analyses of the cell-type-specific expressed genes were conducted in Metascape (http://metascape.org/, accessed on 1 January 2021) [109].

### 4.13. Quantification and Statistical Analysis

Data for quantification analyses are presented as mean ± SD as indicated in the figure legends. The statistical analyses were performed by unpaired two-tailed Student’s *t*-test. The number of biologically independent replicates is shown in the figure legends or figures.

### 4.14. Accession Numbers

The accession numbers for some of the selected genes are as follows: AT5G08330 (TCP21), AT3G53800 (FES1B), AT3G54620 (bZIP25), AT3G58750 (CSY2), AT3G62420 (bZIP53), AT1G32550 (FDC2), AT3G11340 (UGT76B1), AT2G28110 (FRA8/IRX7), AT4G12970 (STOMAGEN/EPFL9), AT1G12920 (ERF1-2), AT2G42790 (CSY3), AT4G37925 (NDHM), AT2G32180 (PTAC18), AT1G69480 (PHO1-H10), AT1G70820, AT5G16030, AT2G29290, AT1G64355, AT5G02590, AT3G48020, AT4G18422, AT3G10530, AT2G35480, AT4G23620, and AT1G04945. ScRNA-seq data are available at the following web address: https://dataview.ncbi.nlm.nih.gov/object/PRJNA577177, accessed on 1 January 2023, with accession numbers SRR11059752, SRR11059753, SRR11059754, and SRR11059755.

## Data Availability

All data supporting the findings of this study are available within the paper and within its Supplementary Data published online.

## Supplementary Materials

The following supporting information can be downloaded at https://www.mdpi.com/article/10.3390/ijms25052553/s1.
