# Supplementary material for: Identification of bZIP Transcription Factors That Regulate the Development of Leaf Epidermal Cells in Arabidopsis thaliana by Single-Cell RNA Sequencing
Source: Int J Mol Sci. 2024 Feb 22;25(5):2553. doi: 10.3390/ijms25052553 (PMC10931525; doi:10.3390/ijms25052553)
Supplement: Supplementary file 1 [file ijms-25-02553-s001.zip › ijms-2843778-supplementary Figures-IJMS.pdf]

Supporting Information

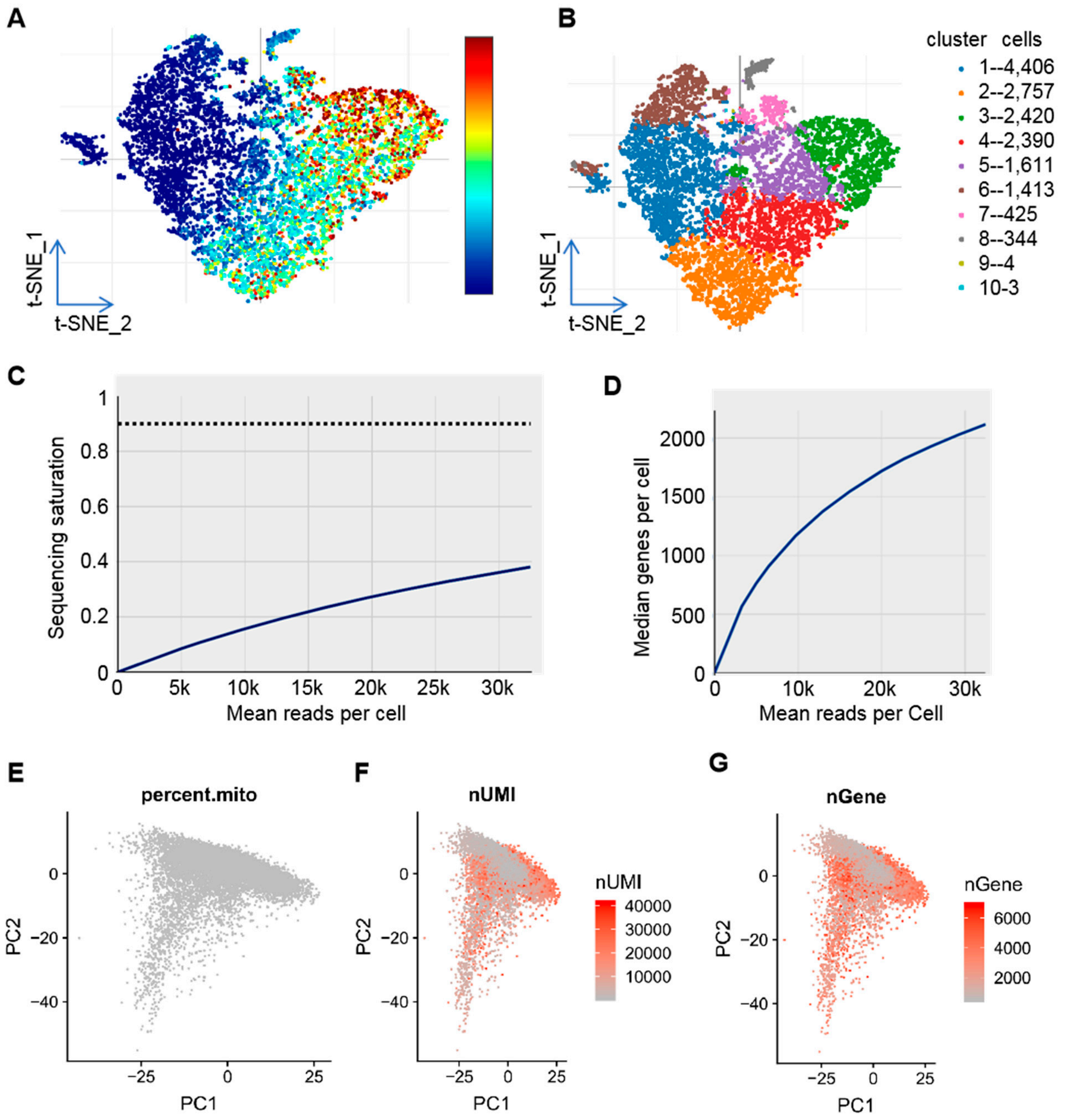

Figure S1. Analysis of the single-cell RNA-sequencing (scRNA-seq) raw data. (A) Total unique molecular identifier (UMI) counts for each cell barcode. Cells with larger UMI counts likely had a higher RNA content than cells with a lower UMI count. Axes correspond to the two-dimensional embedding produced by the tSNE algorithm. (B) Assignments of each cell-barcode cluster by an automated clustering algorithm. The clustering is based on cells having similar expression profiles. The axes correspond to the two-dimensional embedding produced by the tSNE algorithm. (C) Sequencing saturation metric as a function of sequencing depth in mean reads per cell, up to the observed sequencing depth. The slope of the curve near the endpoint can be interpreted as an upper limit to be gained from increasing the sequencing depth beyond this point. The dotted line was drawn at a value that reasonably approximates the saturation point. (D) Median genes per cell as a function of down sampled sequencing

depth in mean reads per cell, up to the observed sequencing depth. The slope of the curve near the endpoint can be interpreted as an upper limit to be gained from increasing the sequencing depth beyond this point. (E) Principal component analysis (PCA) analysis of the percent of mitochondrial gene sequences (percent. mito). (F) PCA analysis of the nUMI (number of UMI). (G) PCA analysis of the number of genes (nGene).

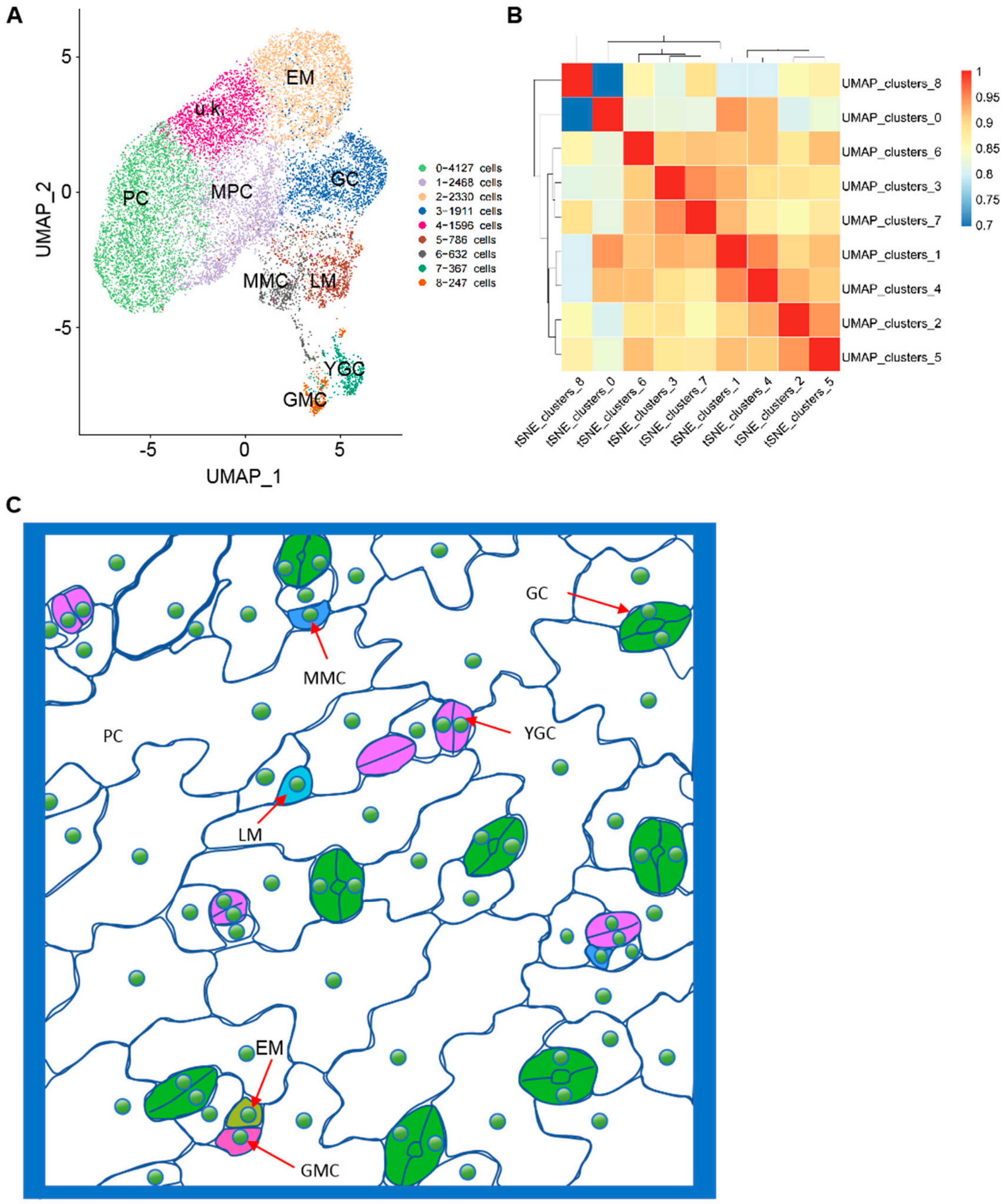

Figure S2. Comparative analysis of the cell clusters identified with different methods of dimensionality reduction. (A) Visualization of the cell clusters derived using the uniform manifold approximation and projection (UMAP) algorithm. Cell clusters identified by tSNE are marked on the UMAP plot. The u.k._10 cells are marked with a circle. (B) Pearson correlation analysis between tSNE and UMAP was performed with the corresponding cell clusters. Color scale: color represents the correlation between rows and columns, with the redder the color, the stronger the positive correlation. (C) Illustration of a leaf surface pattern diagram with different cell types. EM, early-stage

meristemoid; GC, guard cell; PC, pavement cell; LM, late-stage meristemoid; YGC, young guard cell; GMC, guard mother cell; MMC, meristemoid mother cell.

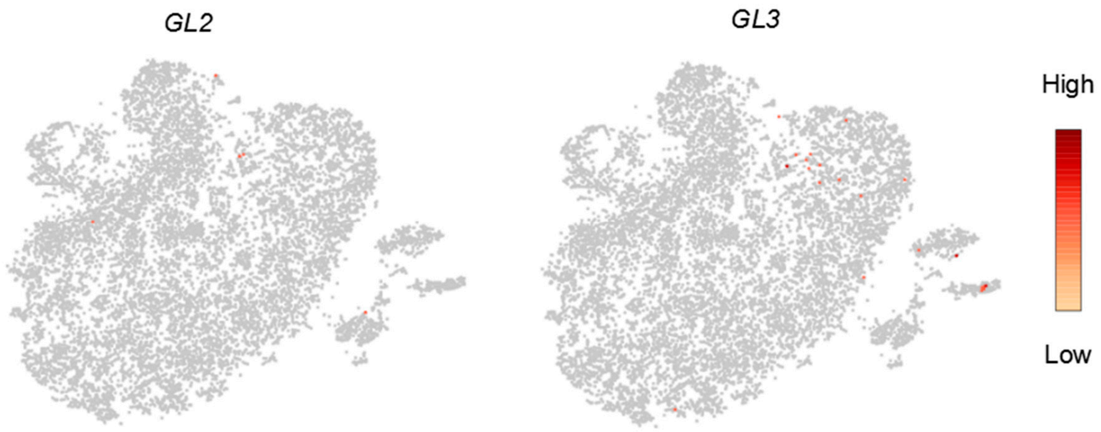

Figure S3. The expression patterns of *GL2* and *GL3* genes. Feature plots of the expression distribution of *GL2* and *GL3*.

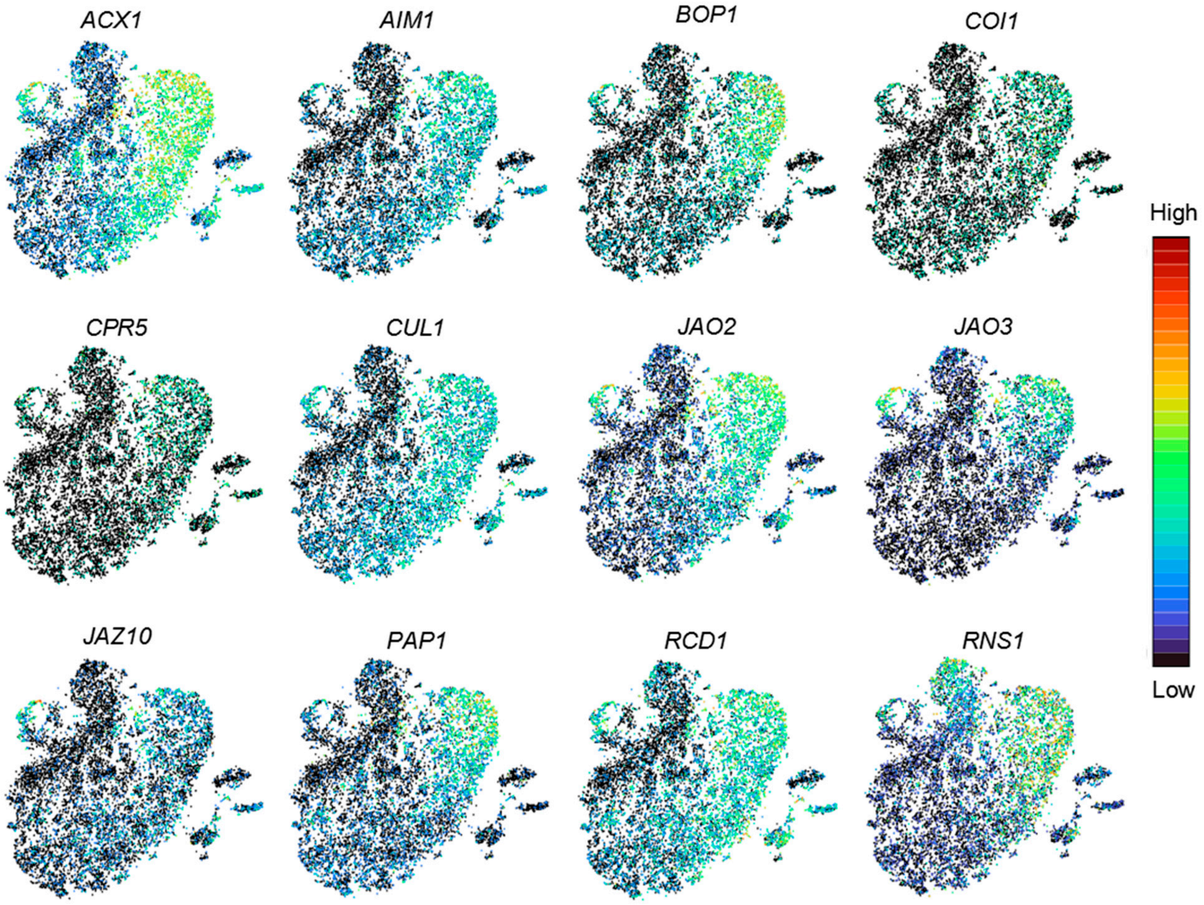

Figure S4. Feature plots of the expression distribution of selected marker genes related to the JA-signaling pathway. Expression levels for each cell are color-coded and superimposed on the tSNE plot. ***ACX1, ACYL-COA OXIDASE 1; AIM1, ABNORMAL INFLORESCENCE MERISTEM; BOP1, BLADE ON PETIOLE1; COI1, CORONATINE INSENSITIVE 1; CPR5, CONSTITUTIVE EXPRESSION OF PR GENES 5; CUL1, CULLIN 1; JAO2, JASMONATE-INDUCED OXYGENASE2; JAO3, JASMONATE-INDUCED OXYGENASE3; JAZ10, JASMONATE-ZIM-DOMAIN PROTEIN 10; PAP1, PRODUCTION OF ANTHOCYANIN PIGMENT 1; RCD1, RADICAL-INDUCED CELL DEATH1; RNS1, RIBONUCLEASE 1.***

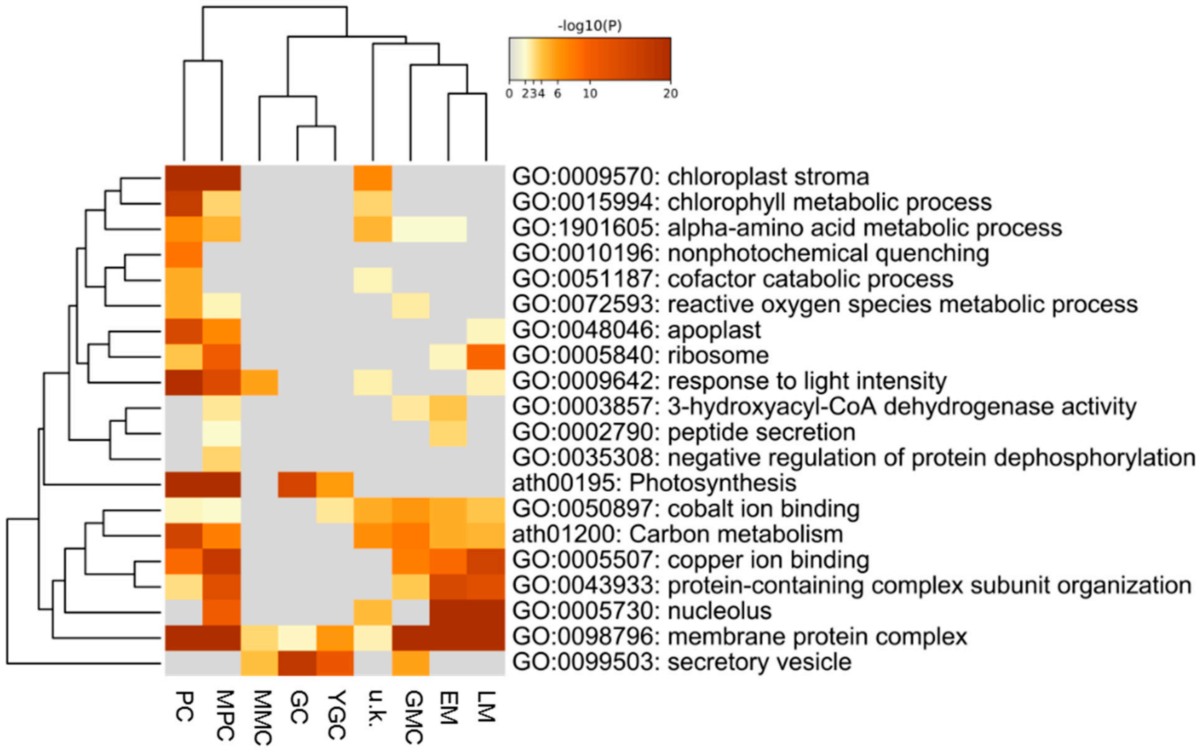

Figure S5. GO and KEGG analyses of cell type-specific expressed genes in different clusters. EM, early-stage meristemoid; GC, guard cell; PC, pavement cell; LM, late-stage meristemoid; YGC, young guard cell; MPC, mesophyll cell; GMC, guard mother cell; MMC, meristemoid mother cell; u.k., unknown.

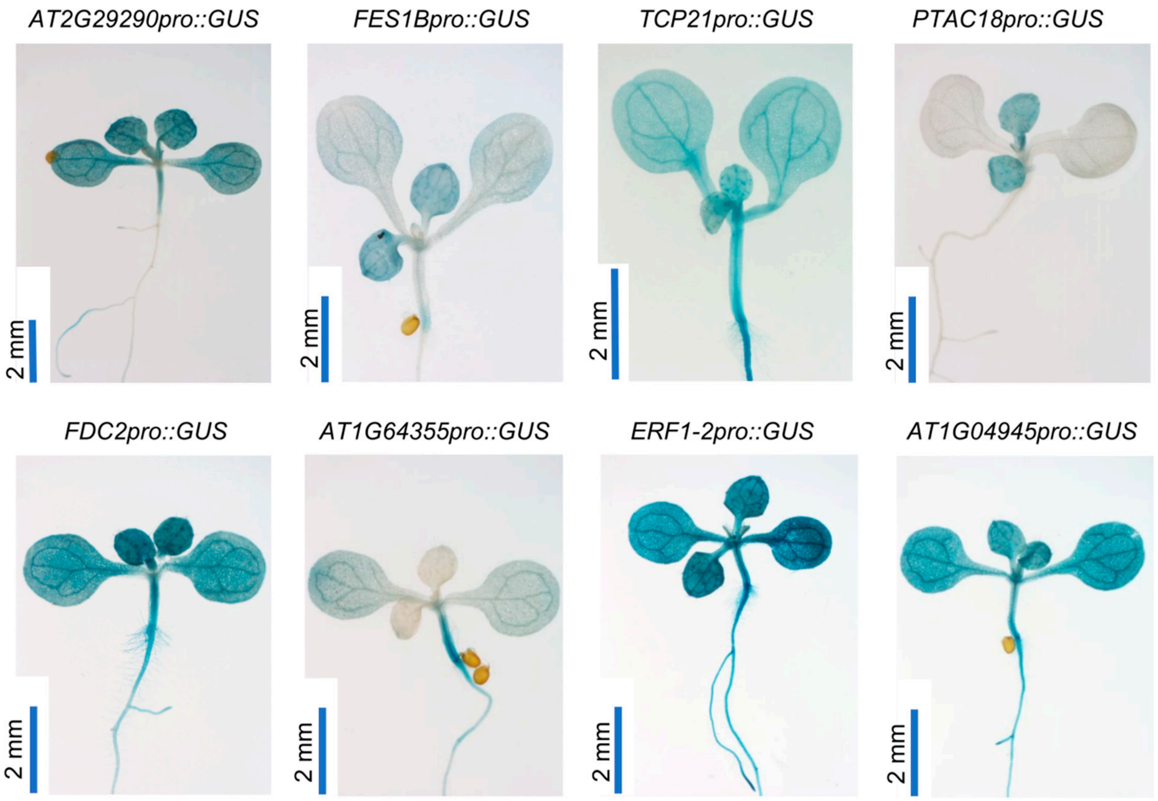

Figure S6. Expression of selected cell type-specific expressed genes in seedlings. Transgenic plants expressing the *GUS* reporter gene driven by the promoters of the selected marker genes were generated to analyze their expression patterns. GUS signals were detected by staining. Scale bar of 2 mm is shown as a blue line.

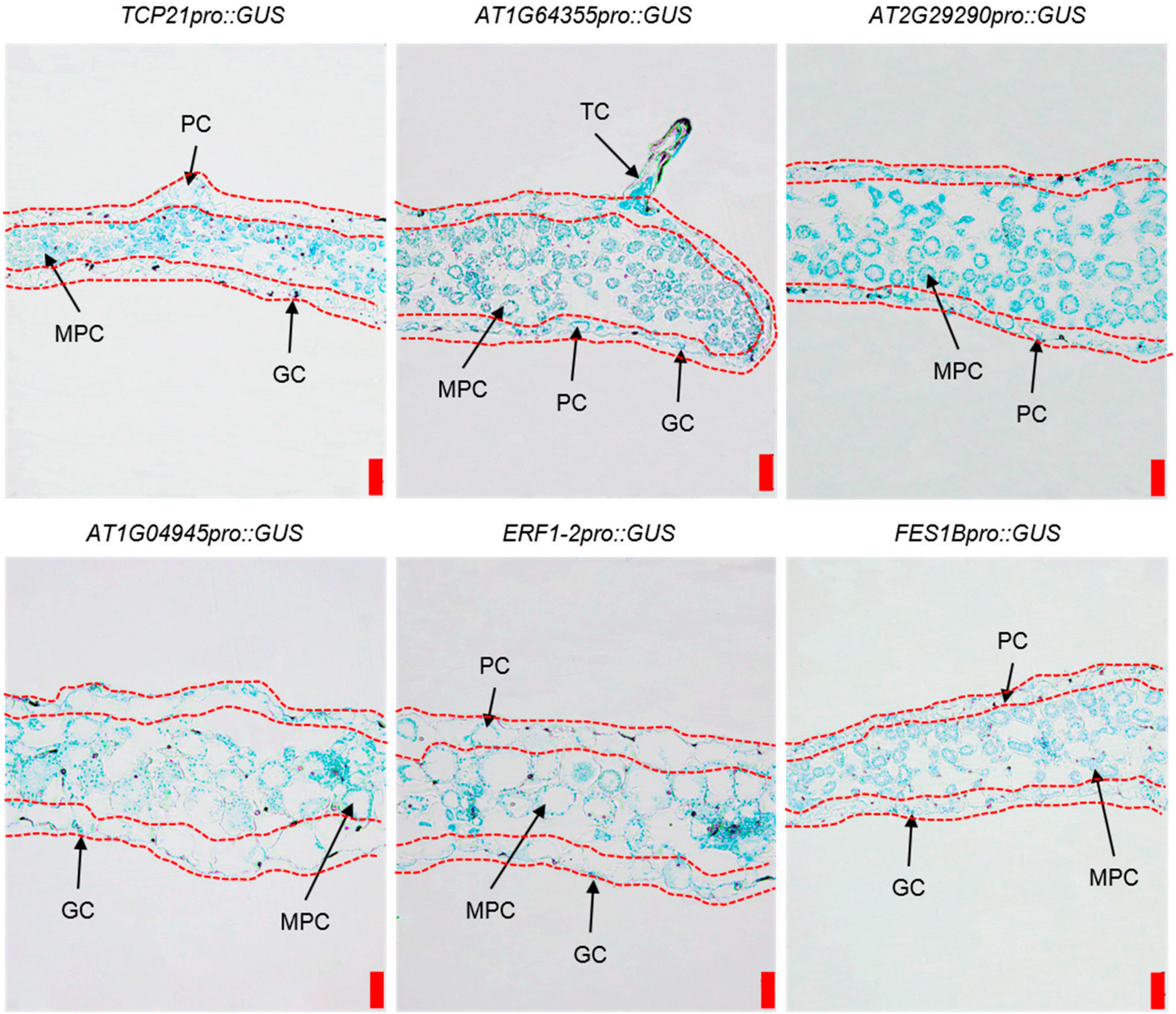

Figure S7. Analysis of the expression patterns of cell type-specific expressed genes. Leaf cross section demonstrates the expression of *GUS* in the *TCP21pro::GUS*, *AT1G64355pro::GUS*, *AT2G29290pro::GUS*, *AT1G04945pro::GUS*, *ERF1-2pro::GUS* and *FES1Bpro::GUS* transgenic plants. The cells in the epidermis are circled with red dot lines. Scale bar, 50 μm. GC, guard cell; MPC, mesophyll cell; PC, pavement cell; TC, trichomes cell.

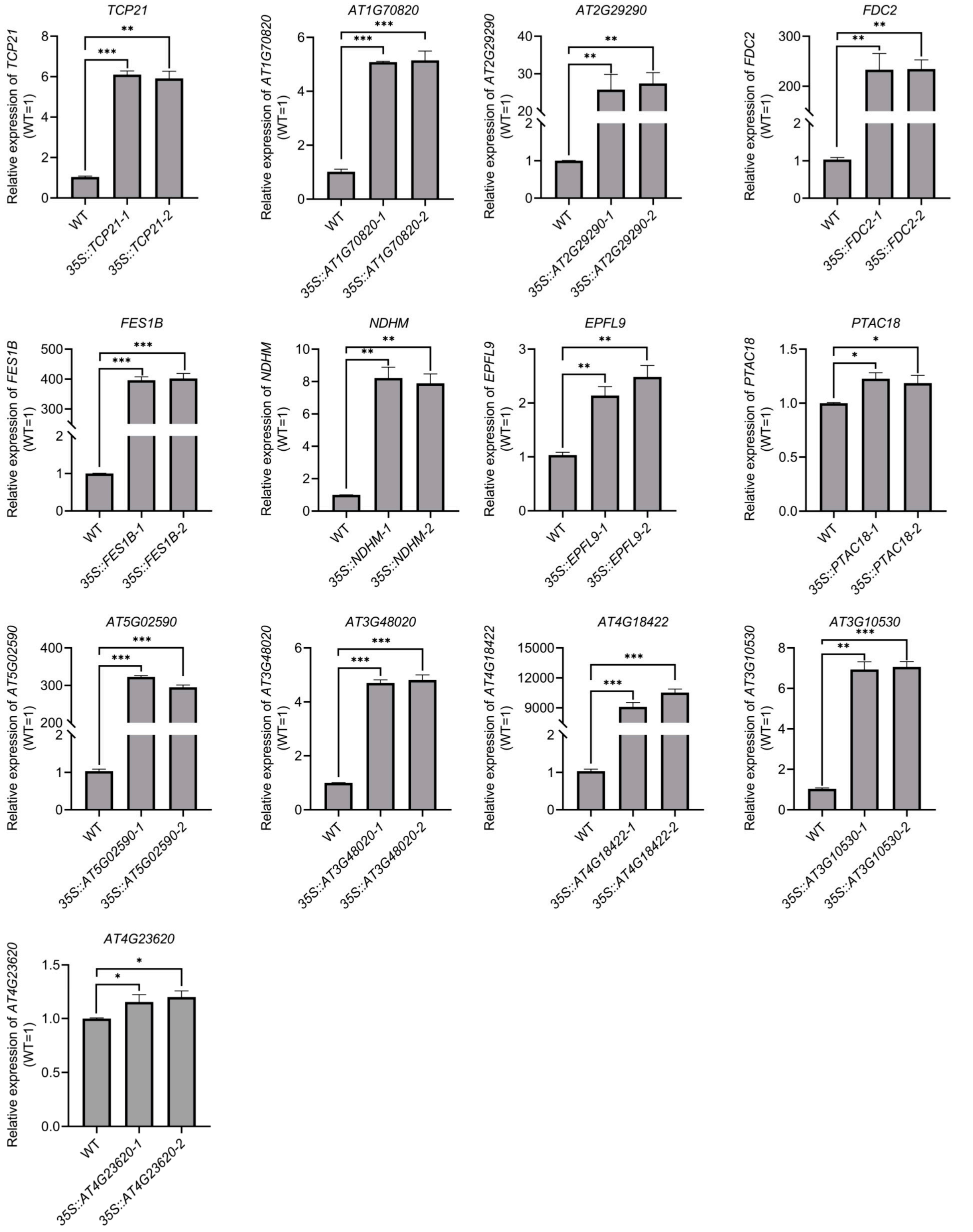

Figure S8. Expression of target genes in transgenic lines. Two representative lines for each target gene were used in this study, and the expression levels of target genes in corresponding transgenic lines were examined by qRT-PCR.

Data represent means ± SDs (n = 3). Asterisks indicate significant difference between each transgenic line and wild type (WT) as determined by a Student's t-test. $^{**}P < 0.01$, and $^{***}P < 0.001$.

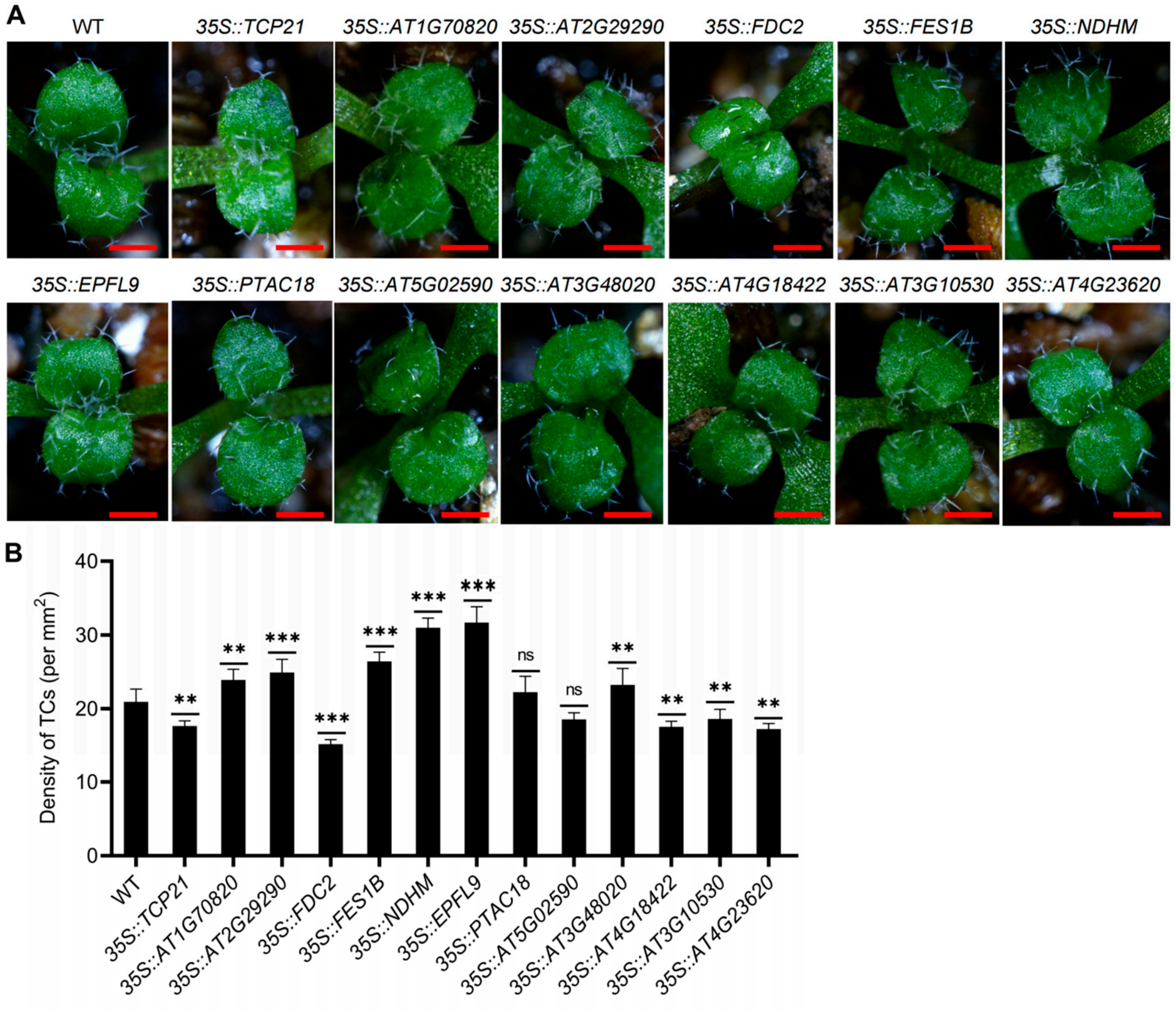

Figure S9. Characterization of the potential roles of selected marker genes for trichome cells (TCs). (A) Detection of the developmental status of trichomes in transgenic and wild-type (WT) seedlings. Scale bar (0.5 mm) is shown as a red line. (B) TC density in the upper epidermis of two 3-day-old true leaves of WT and transgenic seedlings. Data represent means ± SDs (n = 3). Asterisks indicate significant difference between each transgenic line and WT as determined using a Student's t-test. $^{*}P < 0.05$, $^{**}P < 0.01$, and $^{***}P < 0.001$. ns, non-significant.

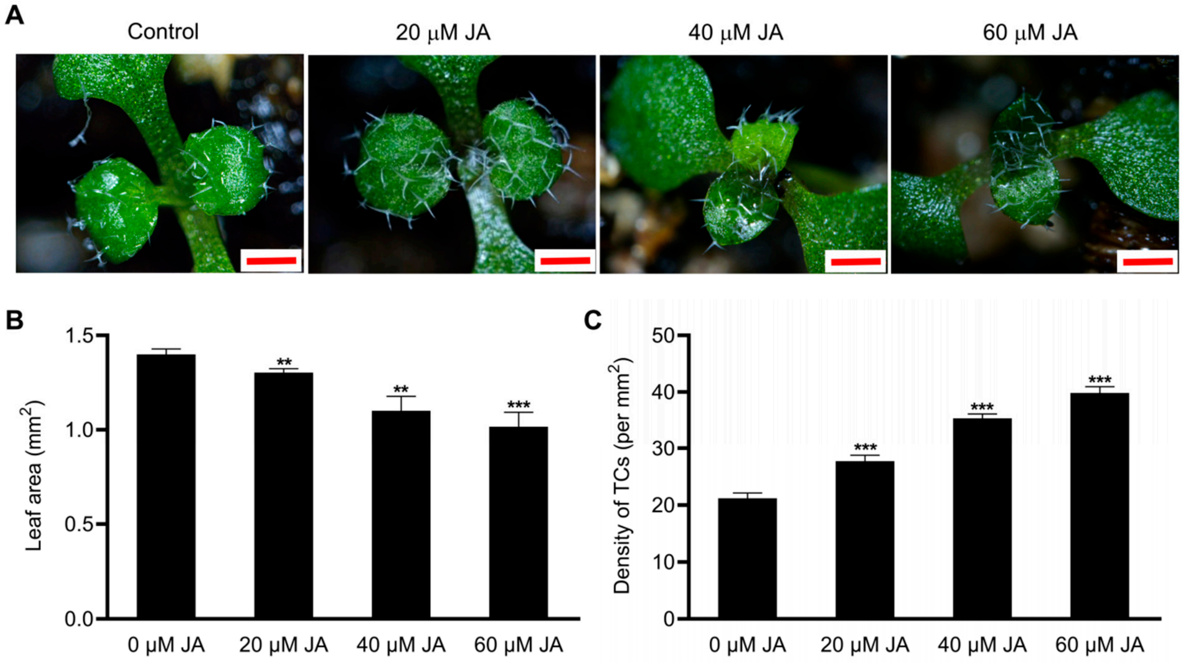

Figure S10. Jasmonic acid (JA) promotes the development of trichome cells (TCs). (A) Developmental status of TCs in the upper epidermis of 3-day-old true leaves of wild-type (WT) plants treated with different concentrations of JA. The developmental status of TCs was photographically recorded. Scale bar of 0.5 mm is indicated as a red line with a white background in the lower right of each photo. (B) Leaf areas of 3-day-old true leaves in WT plants treated with different concentrations of JA. (C) TC densities in the upper epidermis of 3-day-old true leaves of WT seedlings treated with different concentrations of JA. Data represent means ± SDs (*n* = 3). Asterisks indicate significant difference between each JA treatment and control (0 μM JA) as determined using a Student's *t*-test. $^{**}P <$ 0.01, and $^{***}P < 0.001$. ns, non-significant.

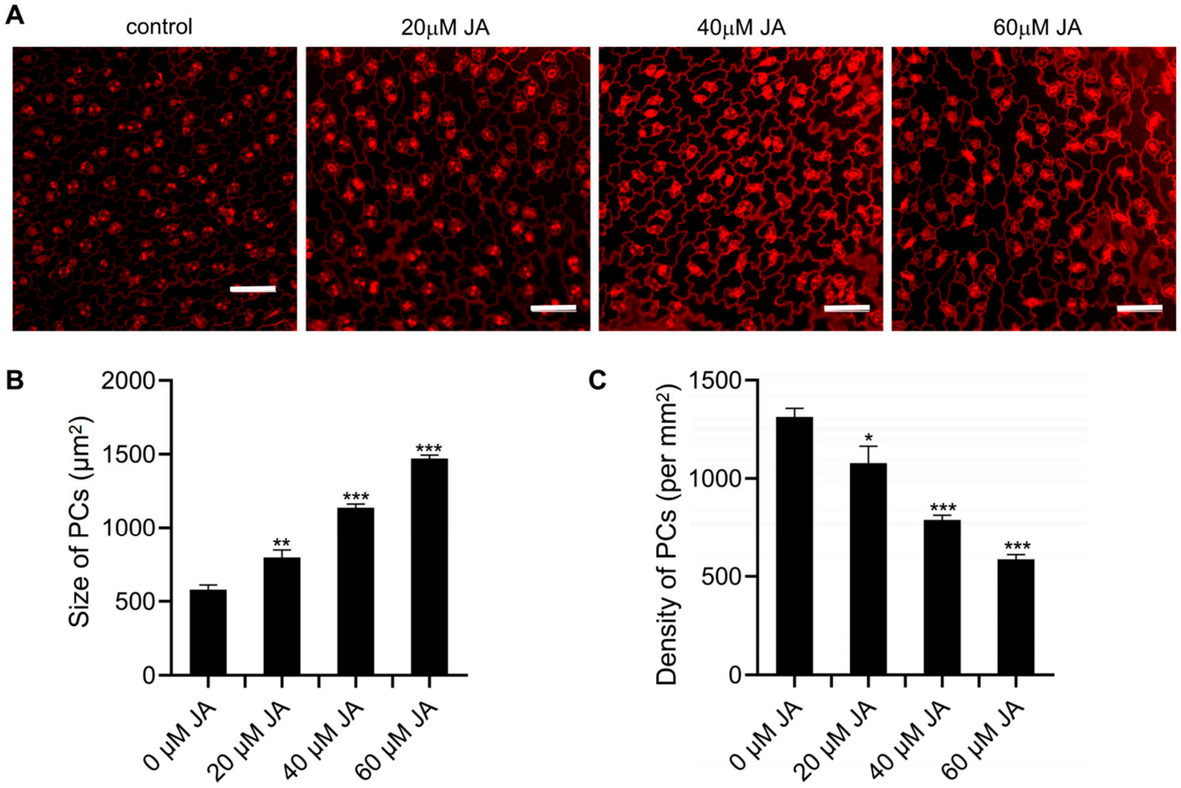

Figure S11. Jasmonic acid (JA) inhibits the development of pavement cells (PCs). (A) Developmental status of PCs in the upper epidermis of 3-day-old true leaves of wild-type (WT) plants treated with different concentrations of JA. The developmental status of PCs was photographically recorded. Scale bar of 50 μm is indicated as a white line in the lower right of each photo. The samples were treated with propidium iodide (PI) for staining to show the cell wall. (B) Sizes of PCs in 3-day-old true leaves in WT plants treated with different concentrations of JA. (C) Densities of PCs in the upper epidermis of 3-day-old true leaves of WT seedlings treated with different concentrations of JA. Data represent means ± SDs (*n* = 3). Asterisks indicate significant difference between each JA treatment and control (0 μM JA) as determined using a Student's *t*-test. $^{*}P < 0.05$, $^{**}P < 0.01$, and $^{***}P < 0.001$. ns, non-significant.

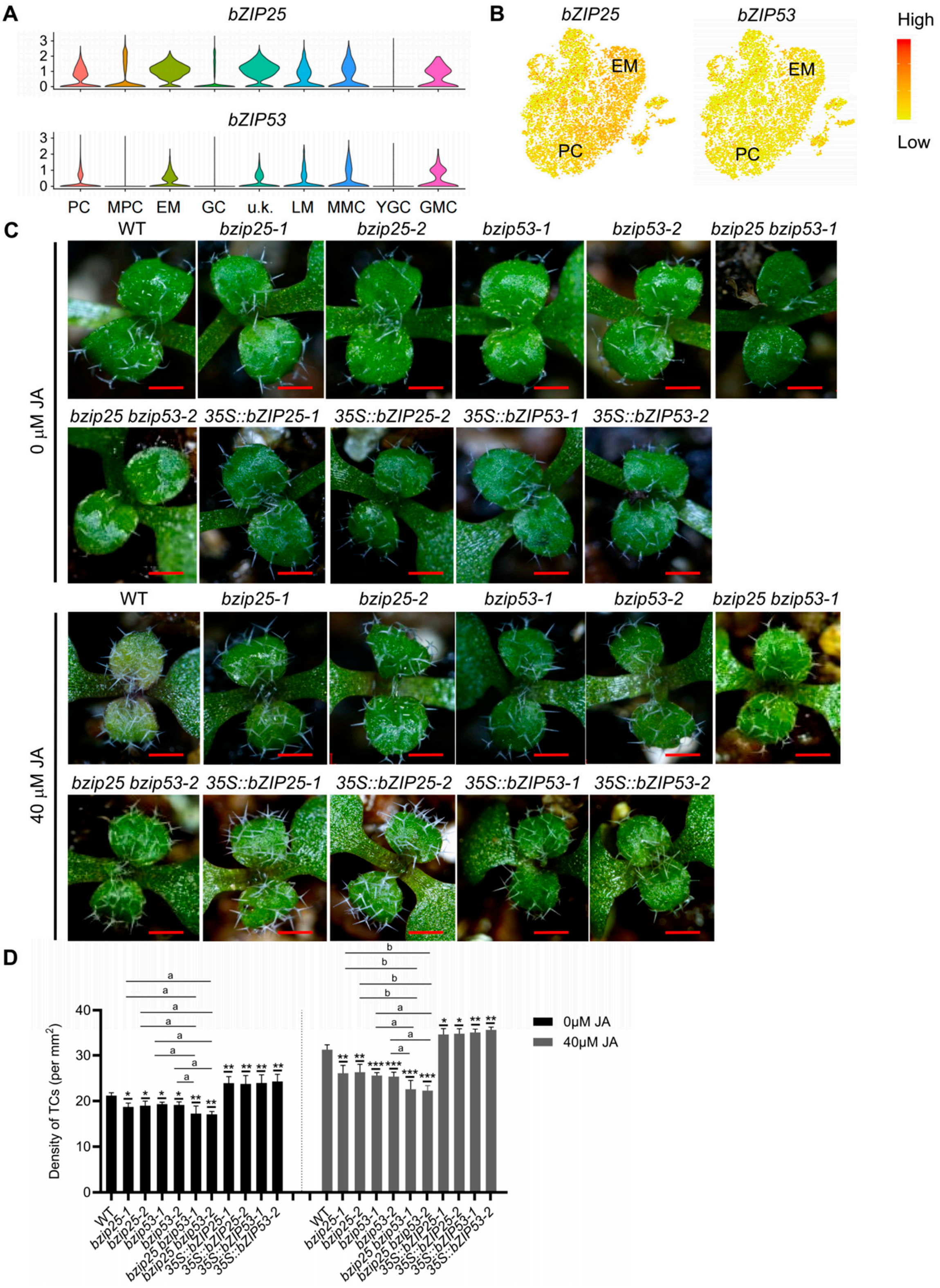
A
bZIP25
bZIP53
PC
MPC
EM
GC
u.k.
LM
MMC
YGC
GMC
B
bZIP25
bZIP53
EM
PC
High
Low
C
0 μM JA
40 μM JA
WT
bzip25-1
bzip25-2
bzip53-1
bzip53-2
bzip25 bzip53-1
bzip25 bzip53-2
35S::bZIP25-1
35S::bZIP25-2
35S::bZIP53-1
35S::bZIP53-2
D
Density of TCs (per mm2)
0μM JA
40μM JA

Figure S12. bZIP25 and bZIP53 positively regulate trichome cell (TC) development. (A) Violin plots showing the expression of *bZIP25* and *bZIP53* in various cell types, including early-stage meristemoid (EM) cells and pavement cells (PCs). (B) Feature plots showing the expression of *bZIP25* and *bZIP53* in EM cells and PCs. (C) Representative photographs of the upper epidermis of 3-day-old true leaves of WT, *bzip25-1*, *bzip25-2*, *bzip53-1*, *bzip53-2*, *bzip25 bzip53*, *35S::bZIP25* and *35S::bZIP53* plants subjected to 0 (control) and 40 μM jasmonic acid (JA) treatments. Bar, 500 μm. (D) Densities of TCs in the upper epidermis of two 3-day-old true leaves of WT, *bzip25-1*, *bzip25-2*, *bzip53-1*, *bzip53-2*, *bzip25 bzip53*, *35S::bZIP25* and *35S::bZIP53* plants subjected to 0 and 40 μM JA treatments. Data represent means ± SDs (n = 3). Asterisks indicate significant difference between each mutant or transgenic line and WT as determined using a Student's t-test. $^{*}P < 0.05$, $^{**}P < 0.01$, and $^{***}P < 0.001$. ns, non-significant. Letters indicate significant difference between each single mutant and double mutant as determined using a Student's t-test. ^{a} $P < 0.05$, ^{b} $P < 0.01$.

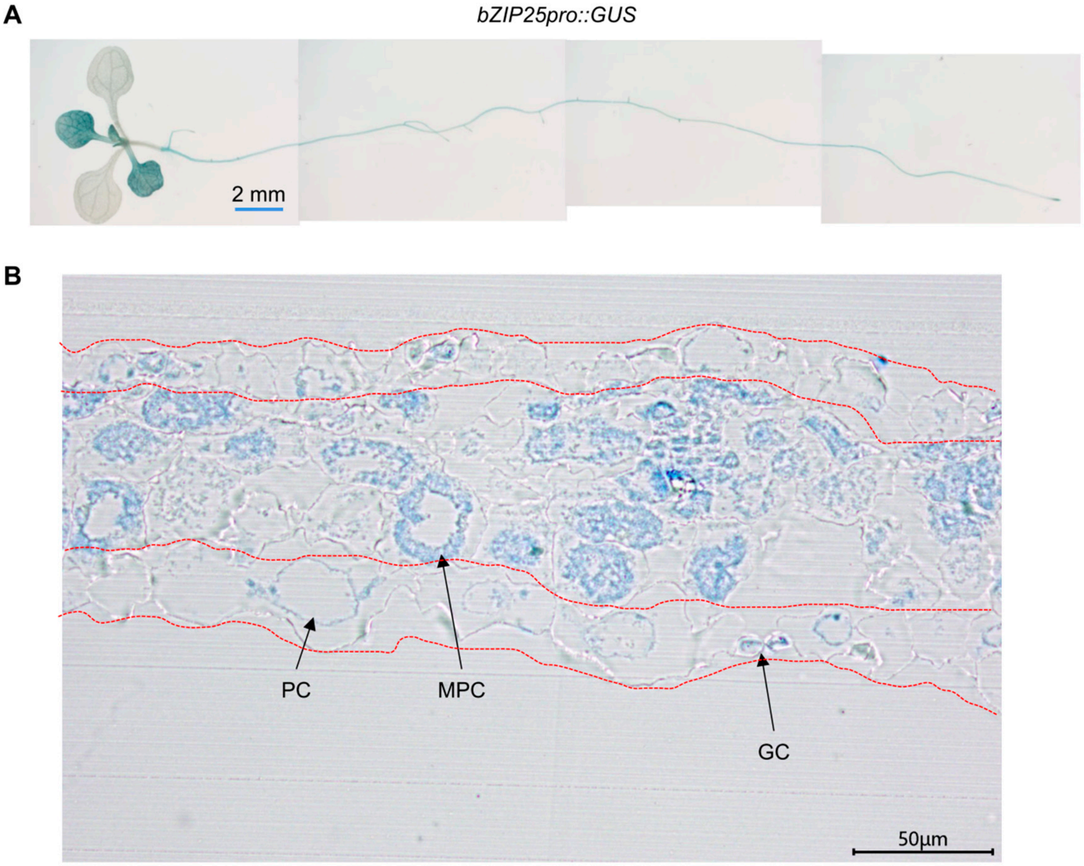

Figure S13. Analysis of the expression patterns of *bZIP25*. (A) Transgenic plant expressing the *GUS* reporter gene driven by the promoter of *bZIP25* was generated to analyze its expression patterns. Scale bar, 2 mm. (B) Leaf cross section demonstrates *GUS* expression in the transgenic plants of *bZIP25pro::GUS*. The cells in the epidermis are circled with red dot lines. Scale bar, 50 μm. GC, guard cell; MPC, mesophyll cell; PC, pavement cell.

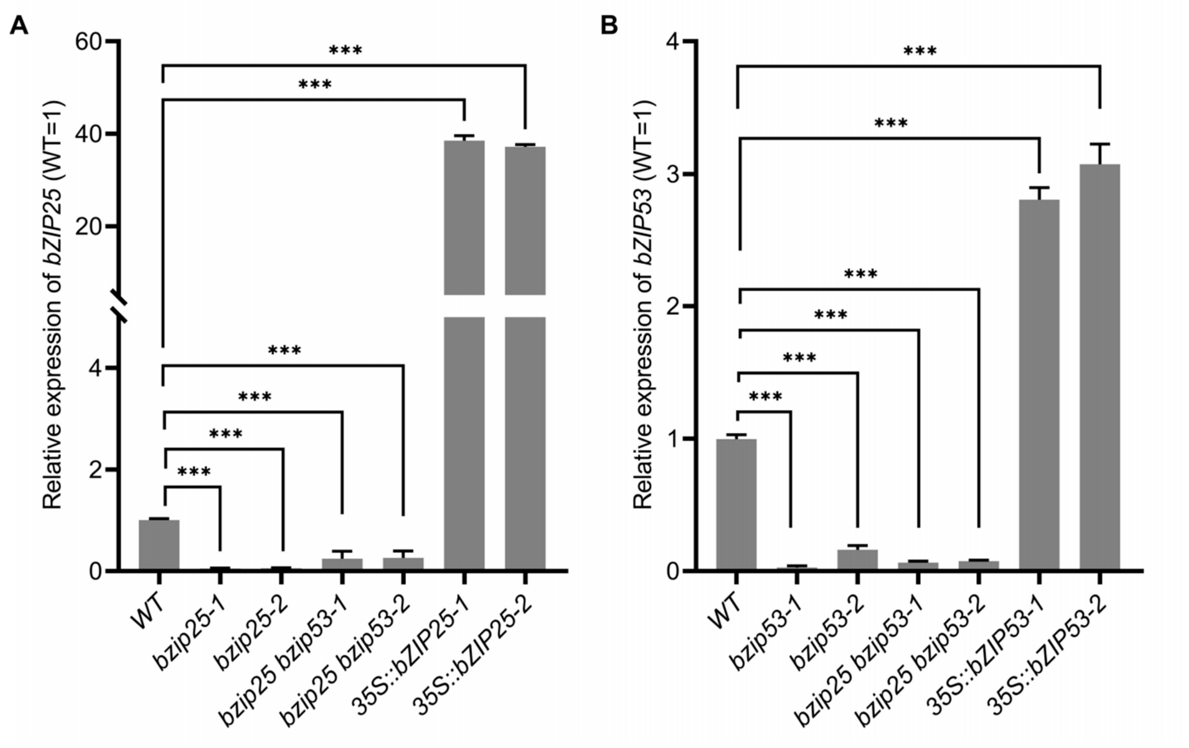

Figure S14. Analysis of the expression levels of *bZIP25* and *bZIP53* in mutants and overexpression lines. (A and B) The expression levels of *bZIP25* (A) and *bZIP53* (B) in studied lines were examined by qRT-PCR. Data represent means ± SDs (n = 3). Asterisks indicate significant difference between each mutant/transgenic lines and wild-type (WT) as determined by a Student's t-test. $^{**}P < 0.01$, and $^{***}P < 0.001$.

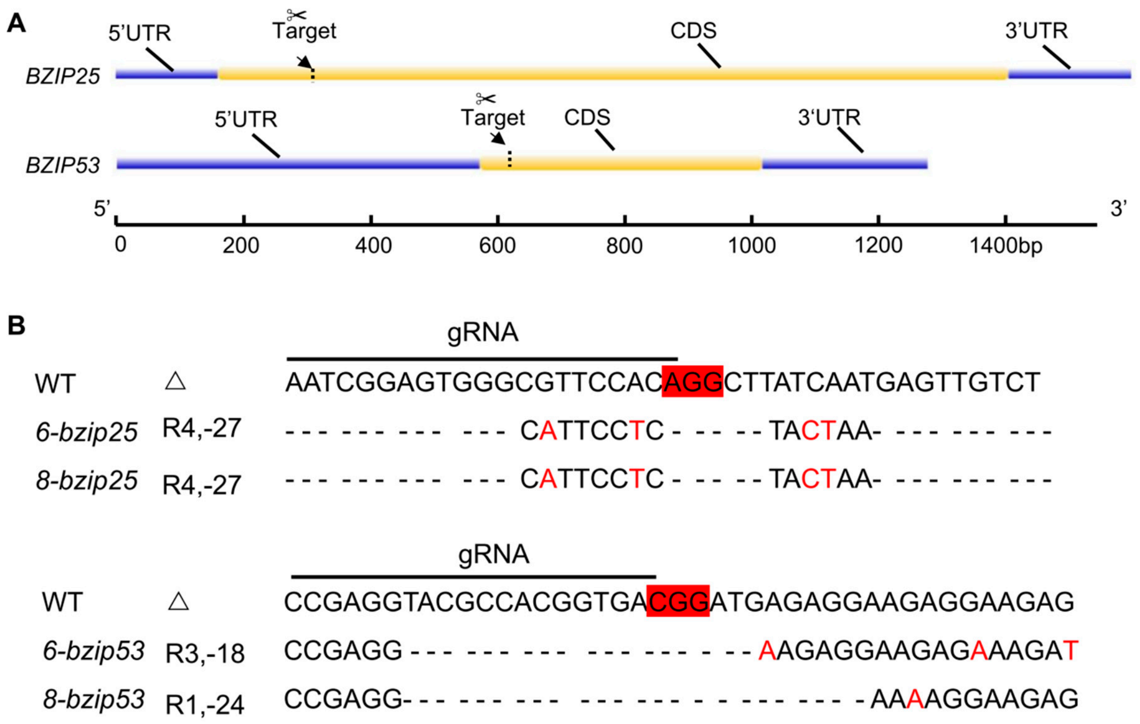

Figure S15. Analysis of the DNA sequences in *bzip25* and *bzip53* seedlings after DNA editing. (A) Diagrammatic illustration of *bZIP25* and *bZIP53* organization: 5'-untranslated region (5'UTR), coding sequences (CDS, exon), 3'-untranslated region (3'UTR), and the target sites are shown. (B) Results of gene editing of *bZIP25* and *bZIP53* were detected by sequencing. *bZIP25* and *bZIP53* sequence results of wild-type (WT) and two representative gene-edited lines (lines 6 and 8). 'R' represents substitution of the base, ' - ' represents a nucleotide deletion. gRNA, guide RNA.

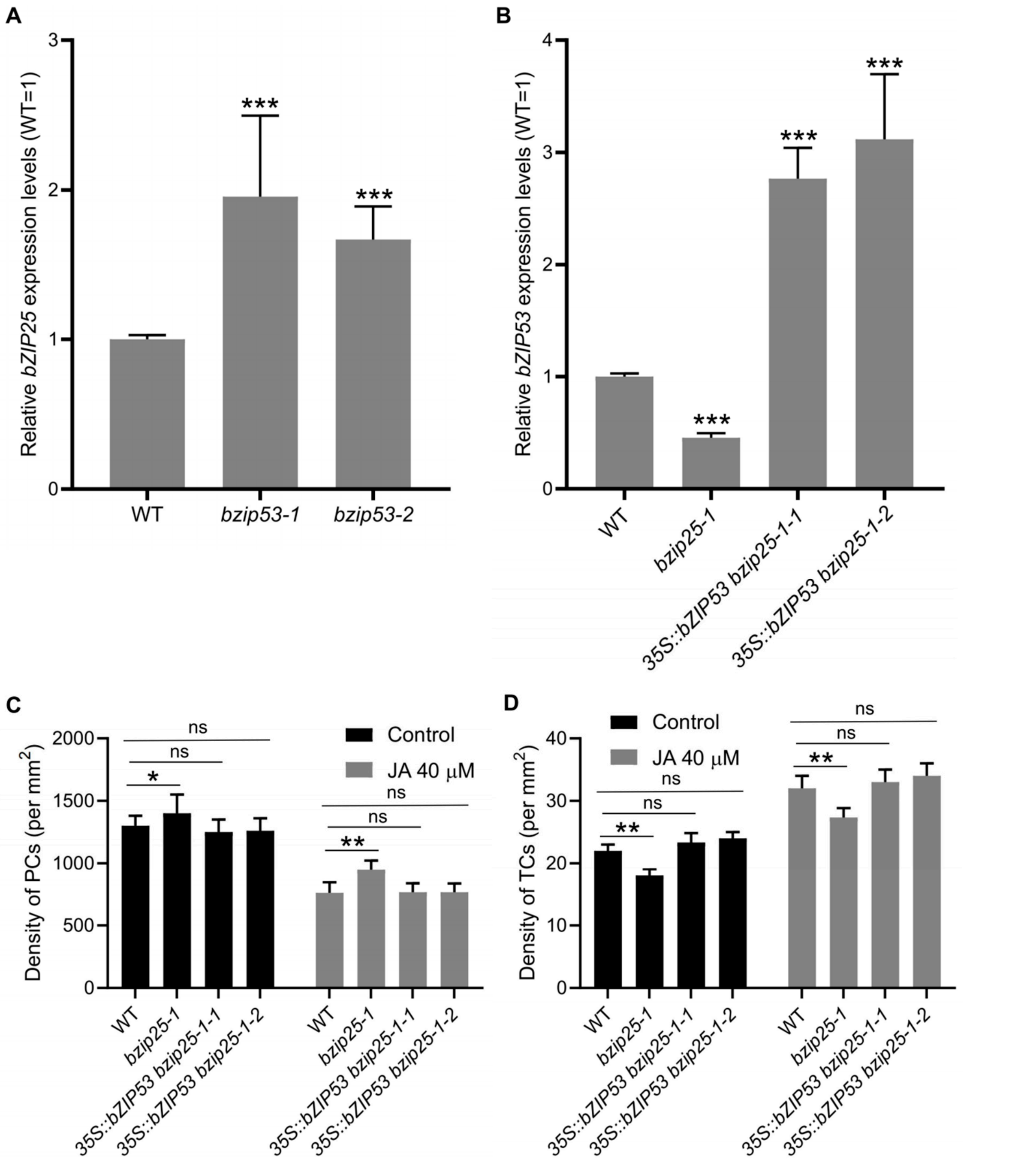

Figure S16. Complementation assay of *bzip25* mutant in the development of pavement cells (PCs) and and trichome cells (TCs) using the cDNA of *bZIP53*. (A) qRT-PCR analysis of the expression of *bZIP25* in *bzip53-1*, *bzip53-2* and wild-type (WT) plants. (B) qRT-PCR analysis of the expression of *bZIP53* in *bzip25-1*, *35S::bZIP53 bzip25-1-1*, *35S::bZIP53 bzip25-1-2* and WT plants. (C-D) Densities of PCs (C) and TCs (D) in the upper epidermis of 3-day-old true leaves of *bzip25-1*, *35S::bZIP53 bzip25-1-1*, *35S::bZIP53 bzip25-1-2* and WT seedlings with (40 μM) and without (Control, 0 μM) jasmonic acid (JA) treatments. Data represent means ± SDs (n = 3). Asterisks indicate significant difference between each mutant or transgenic line and WT as determined using a Student's *t*-test. $^{*}P < 0.05$, $^{**}P <$ 0.01, $^{***}P < 0.001$, and ns, non-significant.

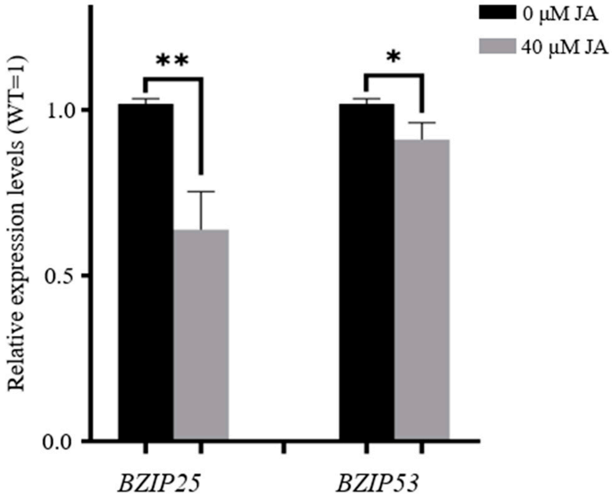

Figure S17. Jasmonic acid (JA) affects the expression of *bZIP25* and *bZIP53* genes. Decreased expression levels of *bZIP25* and *bZIP53* in wild-type (WT) plants after JA treatment. Data represent means ± SDs (n = 3). Asterisks indicate significant difference between JA treatment (40 μM) and control (0 μM). $^{*}P < 0.05$ and $^{**}P < 0.01$.

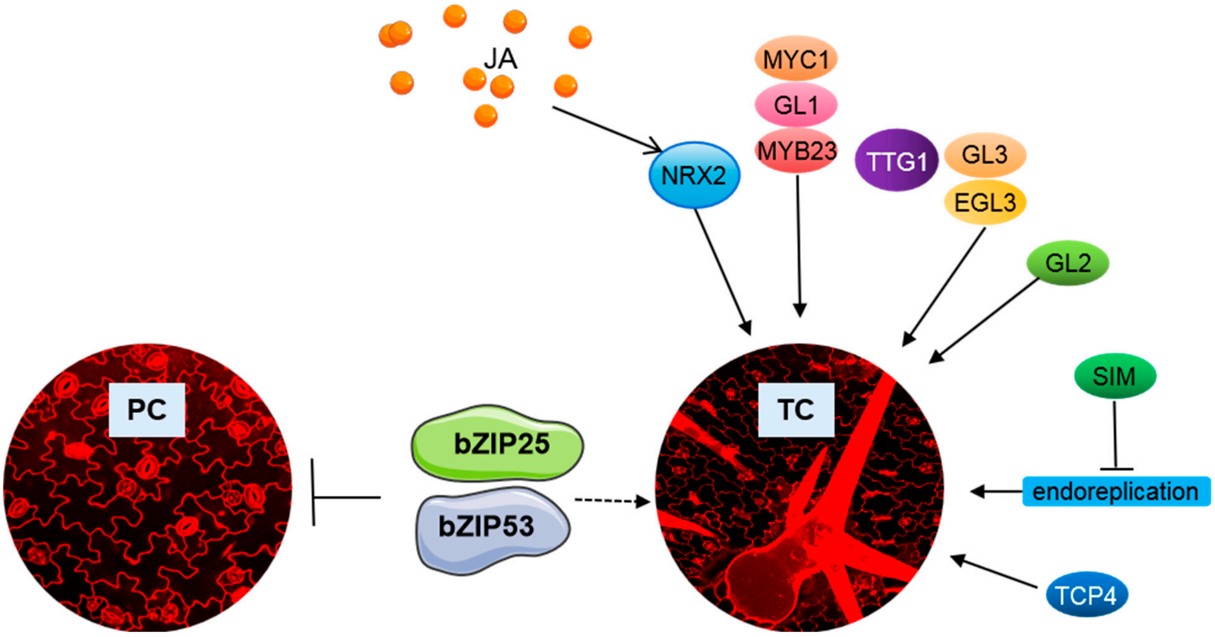

Figure S18. Model of the network of transcription factors, including bZIP25 and bZIP53, in regulating trichome cell (TC) and pavement cell (PC) development. The fate of a TCs is regulated by a series of critical transcription factors, including TTG1, GL2, MYC1, GL3, SIM, NRX2 and TCPs, as well as many others. bZIP25 and bZIP53 negatively and positively regulate the fate and differentiation of PCs and TCs, respectively.

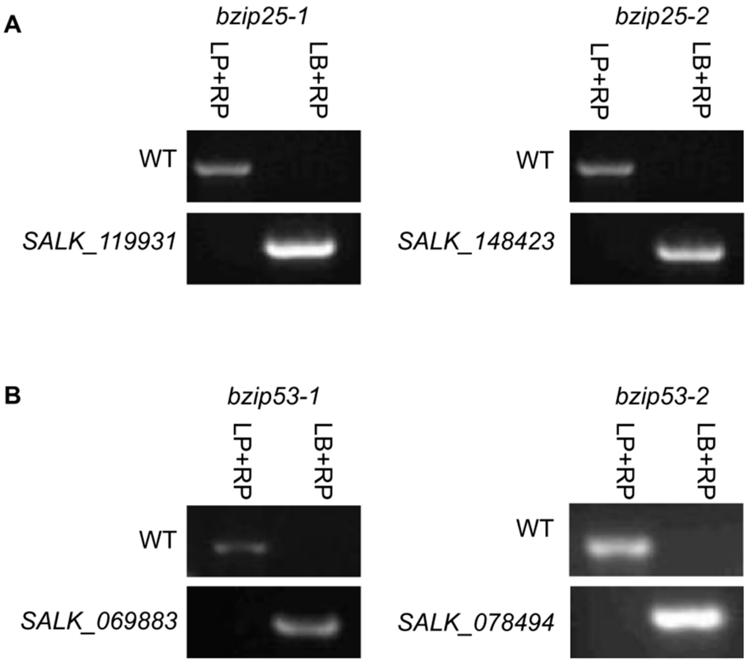

Figure S19. Verification of the T-DNA insertion in mutants by PCR using the corresponding primers.

LP, RP:Left, Right genomic primer; LB: the left T-DNA border primer.
